# Characterization of ApB73, a virulence factor important for colonization of *Zea mays* by the smut *Ustilago maydis*

**DOI:** 10.1111/mpp.12442

**Published:** 2016-08-08

**Authors:** Alexandra Stirnberg, Armin Djamei

**Affiliations:** ^1^ Gregor Mendel Institute (GMI), Austrian Academy of Sciences (OEAW), Vienna Biocenter (VBC) Dr. Bohr‐Gasse 3 Vienna 1030 Austria

**Keywords:** biotrophic interaction, effectors, filamentous fungus, plant pathogen, *Ustilago maydis*, virulence factors, *Zea mays*

## Abstract

The biotrophic fungus *Ustilago maydis*, the causal agent of corn smut disease, uses numerous small secreted effector proteins to suppress plant defence responses and reshape the host metabolism. However, the role of specific effectors remains poorly understood. Here, we describe the identification of ApB73 (Apathogenic in B73), an as yet uncharacterized protein essential for the successful colonization of maize by *U. maydis*. We show that *apB73* is transcriptionally induced during the biotrophic stages of the fungal life cycle. The deletion of the *apB73* gene results in cultivar‐specific loss of gall formation in the host. The ApB73 protein is conserved among closely related smut fungi. However, using virulence assays, we show that only the orthologue of the maize‐infecting head smut *Sporisorium reilianum* can complement the mutant phenotype of *U. maydis*. Although microscopy shows that ApB73 is secreted into the biotrophic interface, it seems to remain associated with fungal cell wall components or the fungal plasma membrane. Taken together, the results show that ApB73 is a conserved and important virulence factor of *U. maydis* that localizes to the interface between the pathogen and its host *Zea mays*.

## Introduction

Biotrophic pathogens require a living host to complete their life cycle. To accomplish this balancing act, they have evolved a fascinating repertoire of secreted effector molecules that allow them to manipulate their hosts. Some effectors have been shown to directly target and inhibit the host defence machinery (Jones and Dangl, 2006; de Jonge *et al*., 2011). Others take part in an evasion strategy, binding and sequestering putative pathogen‐associated molecular patterns (PAMPs), such as cell wall components, to evade recognition and the induction of host defences (de Jonge *et al*., 2010; Mentlak *et al*., 2012). Depending on the functional task of an effector, it will either play a role in the biotrophic interphase between the pathogen and the host (apoplastic effector) or act as a cytoplasmic effector inside the host cell (Koeck *et al*., 2011).

Despite the vital role of effectors for pathogenicity, the actual deletion of many does not necessarily lead to impaired virulence of the pathogen (Kämper *et al*., 2006; Lindeberg *et al*., 2012; Saitoh *et al*., 2012; Schilling *et al*., 2014). This is believed to be partially a result of functional redundancy; however, it is also possible that certain effectors may only be functional under specific environmental conditions that were not reflected in the narrow range of laboratory test conditions used.

For most putative effector proteins, the sequence similarity among species is rather low and conservation across families is rarely reported (Sperschneider *et al*., 2015). This is probably a result of the high selection pressure on effectors to evade the co‐evolving plant defence system (Jia *et al*., 2000; Jones and Dangl, 2006; Le Roux *et al*., 2015). Despite this high variability at the sequence level, functional effector research indicates that many effectors target conserved hubs in the plant defence network and metabolic branching points (Mukhtar *et al*., 2011). Therefore, functional effector studies can be highly informative about the critical needs of biotrophic pathogens, the key nodes in the plant defence system and their interconnection with plant metabolism.

In the last few decades, *Ustilago maydis* has become a versatile model for the study of biotrophic interactions (Djamei and Kahmann, 2012). Infection with the pathogen causes galls on all aerial parts of its host plant *Zea mays* (Brefort *et al*., 2009; Christensen, 1963). The *U. maydis* genome encodes several hundred putative effector proteins allowing the fungus to accomplish its manipulative task. At the genome level, many are arranged in clusters. However, only four of the 12 clusters result in a reduction in virulence when deleted (Kämper *et al*., 2006).

Previous studies have shown that different *Z. mays* varieties display varying susceptibility to *U. maydis* infection. For example, the Early Golden Bantam (EGB) sweet corn variety has been reported to be more susceptible than field corn to *U. maydis* (Agrios, 1988; White, 1999). The inbred line B73, an important commercial variety (Dolgin, 2009), is a widely used maize accession that has recently been fully sequenced (Schnable *et al*., 2009). Accordingly, numerous data are available regarding the susceptibility of B73 to different pathogens (Wisser *et al*., 2006). However, no study has been performed to date to test its susceptibility to *U. maydis* infection. Independent of the maize cultivar, no gene‐for‐gene interactions have been reported for the *Z. mays*–*U. maydis* pathosystem. Instead, variety‐specific differences in infection frequency and severity are based on an interplay of different host loci (Baumgarten *et al*., 2007).

Genetically, *U. maydis* is closely related to other smut fungi, including *Sporisorium reilianum*, *Sporisorium scitamineum*, *Ustilago hordei*, *Ustilago bromivora* and *Melanopsichium pennsylvanicum*. Among these, *S. reilianum* is of special interest as it is most closely related to *U. maydis* (Stoll *et al*., 2003). In addition, the two smuts infect the same host: *Z. mays*. However, *S. reilianum*, as well as *U. hordei* and *U. bromivora*, are head smuts, meaning that they only cause symptoms in the floral organs of their respective monocotyledonous hosts. *Melanopsichium pennsylvanicum* is the only smut fungus known to induce galls on dicotyledonous plants (Sharma *et al*., 2014).

Despite their differences, all mentioned smut fungi use effectors to establish biotrophy (Doehlemann *et al*., 2009; Schirawski *et al*., 2010; Sharma *et al*., 2014).

Experimental evidence explaining the precise role of these effectors is limited. So far, only five effector proteins from *U. maydis* have been functionally characterized. These include two effectors acting in the biotrophic interphase and three that are translocated into the plant cell. The characterized apoplastic effectors are Pep1 (Protein essential for penetration 1; Doehlemann *et al*., 2009; Hemetsberger *et al*., 2012) and Pit2 (Protein involved in tumours 2; Doehlemann *et al*., 2011; Mueller *et al*., 2013). Pep1 is known to inhibit plant peroxidases and thereby interferes with the oxidative burst, whereas Pit2 inhibits cysteine proteases in the biotrophic interface. The effectors reported to be translocated into the host cell are the secreted chorismate mutase Cmu1 (Djamei *et al*., 2011), a protein rechannelling chorismate into the phenylpropanoid pathway and thereby outcompeting the biosynthesis pathway of the plant defence hormone salicylic acid, Tin2 (Tumour inducing 2; Tanaka *et al*., 2014), which is capable of interfering with lignin biosynthesis in maize by promoting anthocyanin biosynthesis, and See1 (Seedling efficient effector 1; Redkar *et al*., 2015a), the first functionally characterized organ‐specific effector. See1 interacts with a maize homologue of SGT1 (Suppressor of G2 allele of skp1) involved in cell cycle control in yeast.

Here, we characterize ApB73, a protein transcriptionally up‐regulated during the biotrophic stage and essential for the successful colonization of *Z. mays* accession B73 by *U. maydis*. ApB73 is secreted by the fungus, but then localizes to the fungal surface during biotrophy.

## Results

### Discovery of ApB73

**Figure 1:**
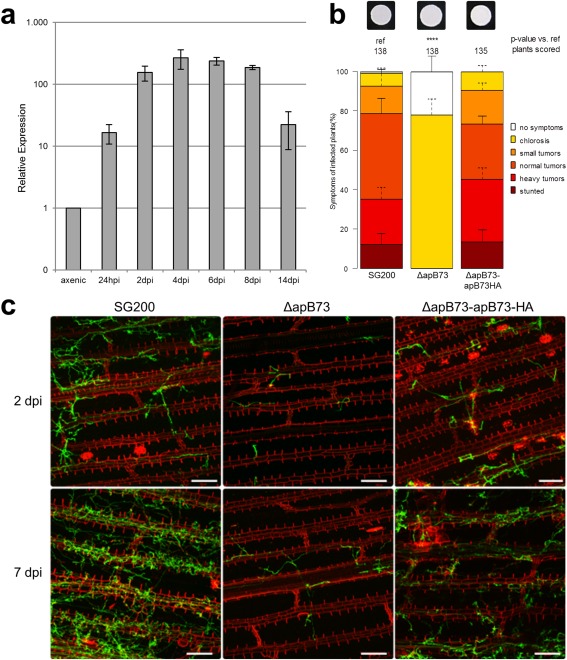
ApB73 is an important virulence factor of *Ustilago maydis*. (a) *apB73* is up‐regulated during biotrophic growth stages. Transcript levels of *apB73* were measured at different growth stages of FB1/FB2 by quantitative real‐time polymerase chain reaction (qPCR). For normalization, the constitutive gene peptidyl‐prolyl isomerase (*ppi*) was used. We analysed three biological replicates; error bars indicate standard deviation. (b) ApB73 is essential for the virulence of *U. maydis* in maize cv. B73. Disease rating of maize seedlings at 12 days post‐infection (dpi) with the progenitor strain SG200, the SG200ΔapB73 deletion strain or the complementation strain SG200ΔapB73‐apB73‐HA. In the top row, photographs of the respective strain are shown after growth on filamentation‐inducing charcoal plates for 24 h. Disease scores are shown on the right. The mean values of three independent infections are depicted and the total number of infected plants is indicated above the respective columns. The mean and standard deviation of relative counts from replicates are displayed. For clarity, only positive error bars are shown. *P* values were calculated by Fisher's exact test. Multiple testing correction was performed using the Benjamini–Hochberg procedure. ****P < 0.0001. (c) Microscopic analysis of the ApB73 virulence phenotype. Maize plants of cv. B73 were infected with the progenitor strain SG200, the SG200ΔapB73 deletion strain or the complementation strain SG200ΔapB73‐apB73‐HA, and harvested at 2 or 7 dpi. Photographs were taken after staining the leaf tissue with WGA‐AlexaFluor488 (green) to visualize fungal chitin and propidium iodide (red) to observe plant cell walls. Bar, 100 µm.

Previously published microarray studies, comparing *U. maydis* transcripts from infected maize tissue with those of axenically grown cultures, demonstrated a strong up‐regulation of transcripts from putative effector genes (Skibbe *et al*., 2010). Among these induced transcripts, we found UMAG_02011 to be up‐regulated during biotrophy. To confirm this observation, we performed quantitative real‐time polymerase chain reaction (qPCR) analysis (Fig. 1a). It showed that UMAG_02011 is transcriptionally up‐regulated by more than 100‐fold during biotrophic growth of the fungus. This indicates that the expression of UMAG_02011 might be specifically required to establish and/or maintain biotrophy.

The intron‐less *apB73* gene (UMAG_02011) is located on chromosome 3 of the *U. maydis* genome and is not part of a gene cluster. The flanking gene upstream is UMAG_02010 (*aro‐8*), which is predicted to be related to the family of II 2‐keto‐3‐deoxy‐d‐arabino‐heptulosonate 7‐phosphate synthases which are involved in amino acid metabolism. The next coding sequence downstream of *apB73* is UMAG_02012, a hypothetical protein that may be involved in sugar, glucoside, polyol and carboxylate metabolism. Both proteins are not predicted to be secreted, in contrast to ApB73, which is expected to be cleaved after a 19‐amino‐acid‐long N‐terminal putative secretion signal/signal peptide (SP; Petersen *et al*., 2011).

With a size of 709 amino acids ApB73 is larger than the common size ranges of an effector protein. Despite its size, there are no known protein domains within ApB73, apart from the SP. Possible orthologues of ApB73 can be found in related smut fungi, including *S. reilianum*, *S. scitamineum*, *U. hordei*, *U. bromivora* and *M. pennsylvanicum*. However, none of these proteins has been characterized to date. Interestingly, but similar to many other known *U. maydis* effectors (e.g. Cmu1, Tin2 and Pep1), orthologues can also be found in the genome of several different *Pseudozyma* species, a non‐pathogenic yeast‐like fungus (Buxdorf *et al*., 2013).

### Role of *apB73* for virulence

To elucidate the importance of *apB73*, gene deletions and overexpression strains were generated by homologous recombination in the solopathogenic haploid strain SG200 (Kämper *et al*., 2006). The overexpressor strain was generated by ectopic integration of the *apB73* gene under the strong constitutive *oma* promoter (Flor‐Parra, 2006) into the *ip* locus (Keon *et al*., 1991; Loubradou *et al*., 2001). First, a possible role of ApB73 in non‐infection‐related stress responses was investigated. The deletion strain, as well as an overexpressor, was subjected to different stress‐inducing media. The tested conditions included nutrient depletion, salt, oxidative and cell wall stress. No obvious growth differences were observed in the *apB73* deletion strain or the ApB73 overexpressor strain relative to SG200 (Fig. S2, see Supporting Information). The ability to grow filaments, a prerequisite for the pathogenic lifestyle of *U. maydis* (Kahmann and Kämper, 2004), was tested by plating the progenitor strain and the *apB73* deletion strain on charcoal medium (Fig. 1b). On this filamentation‐inducing medium, SG200ΔapB73 and SG200 form fuzzy white colonies, indicating that the ability to form filaments remains intact (see Fig. S1 for negative control).

In order to assess the role of ApB73 in pathogenicity, seedlings of *Z. mays* cultivar B73 were infected with the solopathogenic strain SG200 and the deletion strain SG200ΔapB73. Disease symptom scoring showed that the mutant strain fails to infect the *Z. mays* cultivar B73, leading to a complete loss of tumour formation (Fig. 1b).

To test for causality between the virulence defect and the absence of *apB73*, the *apB73* deletion strain was complemented by ectopic integration of the *apB73* gene into the *ip* locus. The resulting complementation strain (SG200ΔapB73‐P_apB73_‐apB73‐HA) displays SG200‐like virulence in seedling infection assays on B73 maize plants (Fig. 1b). This confirms that the deletion of *apB73* is the cause of the virulence loss of the SG200ΔapB73 strain on B73 seedlings. The dramatic reduction in the development of macroscopic symptoms in SG200ΔapB73‐infected B73 maize plants could be a result of either the inability of the mutant strain to penetrate epidermal cells, as has been reported for the effector mutant Δ*pep1* (Doehlemann *et al*., 2009), or its inability to proliferate efficiently inside the host plant. To clarify this, microscopic analysis of different fungal infection stages was performed. Seedlings of maize cultivar B73 harvested 2 or 7 days after infection with SG200, SG200ΔapB73 or SG200ΔapB73‐P_apB73_‐apB73‐HA were observed (Fig. 1c). Although SG200ΔapB73 mutant strains still seemed to be able to form appressoria and penetrate, proliferation inside the plant was strongly reduced. The progenitor strain‐like formation of appressoria and penetration structures was independently confirmed by a microscopy‐based assay, comparing appressorial marker induction and penetration efficiency of fluorescently labelled *apB73* mutant and progenitor strains (Fig. S3, see Supporting Information). Similar microscopic virulence phenotypes have been reported previously for the *U. maydis* mutant Δ*pit1*, a non‐secreted integral membrane protein (Doehlemann *et al*., 2011). Summarizing these observations, the *apB73* mutant did not seem to be impaired in its ability to penetrate the plant cuticle and plant cell walls, but appeared to be inhibited in successful colonization of the maize leaf tissue after initial penetration. This is consistent with the finding that tassels of the maize variety Gaspe Flint infected with dikaryotic *apB73* deletion mutants show reduced gall formation in comparison to infection with the control progenitor strain FB1/FB2 (Fig. S4, see Supporting Information). Microscopic analysis revealed that the galls of plants infected with the *apB73* deletion mutant did not contain any spores, in contrast to the galls that were induced by the progenitor strain (Fig. S4).

**Figure 2:**
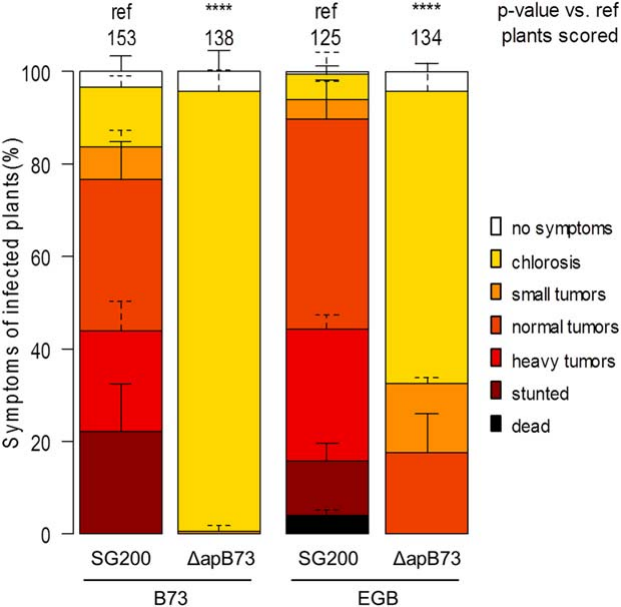
Effect of ApB73 on virulence in different *Zea mays* accessions. Virulence assay showing the different outcomes of disease ratings of the ΔapB73 mutant depending on the use of maize accession B73 or EGB. Seedlings were infected 7 days after germination and scored for symptoms at 12 days post‐infection (dpi). Disease scores are shown on the right. The mean values of three independent infections are depicted and the total number of infected plants is indicated above the respective columns. The mean and standard deviation of relative counts from replicates are displayed. For clarity, only positive error bars are shown. *P* values were calculated by Fisher's exact test. Multiple testing correction was performed using the Benjamini–Hochberg procedure. ****P < 0.0001.

As different maize varieties show varying susceptibility to *U. maydis*, the highly susceptible sweet corn variety EGB was also challenged with SG200ΔapB73 and SG200. Interestingly, and in contrast to B73, EGB plants still show tumour formation upon infection with SG200ΔapB73, but at a significantly lower rate in comparison to infections with SG200 (Fig. 2).

### Secretion of ApB73

**Figure 3:**
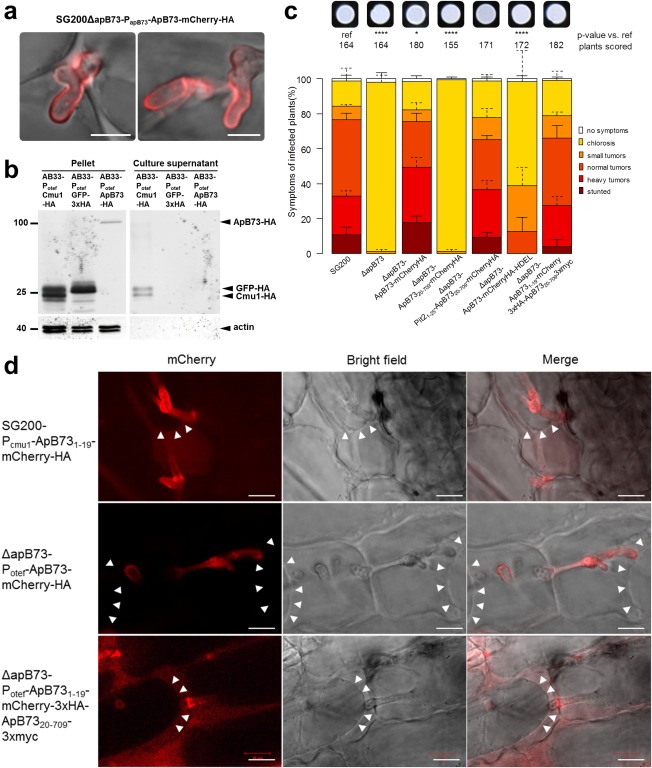
Localization of the ApB73 protein and the role of its signal peptide. (a) Secretion of ApB73‐mCherry‐HA protein in infected maize plants. Leaves of maize cv. B73 were infected with SG200ΔapB73‐ApB73_1–709_‐mCherry‐HA and fluorescence was observed at 5 days post‐infection (dpi). Bar, 5 μm. (b) Detection of ApB73‐HA protein in culture supernatants. ApB73‐HA was expressed by strain AB33‐P_otef_‐ApB73‐HA after shifting the cells to nitrate‐containing medium for 6 h to induce filamentation. AB33‐P_otef_‐Cmu1‐HA was used as a positive control and AB33‐P_otef_‐GFP‐3×HA as a negative control. Supernatants were collected; the proteins present were precipitated with trichloroacetic acid (TCA)/Sodium deoxycholate (DOC) and subjected to Western blot analysis using anti‐haemagglutinin (HA) and anti‐actin antibodies. Compared to Cmu1‐HA, five times more precipitated supernatant‐derived proteins of ApB73‐HA were loaded. Numbers on the left indicate the size in kilodaltons (kDa). (c) Virulence assay confirming the essential role of the ApB73 signal peptide and the complementation of the C‐ and N‐terminal fusions to mCherry. Strains expressing ApB73 without SP (ApB73_20–709_), with a different SP (Pit2_1–25_‐ApB73_20–709_), the endoplasmic reticulum (ER) retention signal HDEL (ApB73_1–709_mCherryHA‐HDEL) or in C‐ or N‐terminal fusion to mCherry were compared to the deletion strain (ΔapB73) and the progenitor strain SG200 in a seedling infection assay using maize cv. B73. All strains were expressed under the native *apB73* promoter, except for the N‐terminal mCherry fusion (*otef* promoter). In the top row, photographs of the respective strain are shown after growth on filamentation‐inducing charcoal plates for 24 h. Disease scores obtained at 12 dpi are shown on the right. The mean values of three independent infections are depicted and the total number of infected plants is indicated above the respective columns. The mean and standard deviation of relative counts from replicates are displayed. For clarity, only positive error bars are shown. *P* values were calculated by Fisher's exact test. Multiple testing correction was performed using the Benjamini–Hochberg procedure.*P < 0.05; ****P < 0.0001. (d) Effect of plasmolysis on the localization of different ApB73 fusion proteins. Maize plants of cv. B73 were infected with strains expressing ApB73_1–19_mCherry‐HA, ApB73‐mCherry‐HA or ApB73_1–19_‐mCherry‐3×HA‐ApB73_20–709_−3×myc, harvested at 3 dpi and observed after treatment with 1 m mannitol. Arrowheads indicate the plant plasma membrane pulling away from the cell wall. Bar, 10 µm.

As a result of the strong disease phenotype of the *ap*B73 deletion mutant, it can be concluded that ApB73 is an important virulence factor of *U. maydis*. Its predicted secretion signal and the lack of any observable phenotype on stress plates implicates that ApB73 might act as an effector. To test for the localization of ApB73 *in planta*, we tagged *apB73* at its C‐terminus with mCherry‐HA and expressed the fusion protein under the native *apB73* promoter. In liquid culture, no mCherry signal was observed. However, during biotrophic development *in planta*, the mCherry signal could be observed all around the hyphae of the fungus. This indicates that *U. maydis* secretes ApB73 into the biotrophic interphase (Fig. 3a). To further confirm the secretion of ApB73 by *U. maydis*, we performed a Western blot on secreted proteins, termed supernatant assay. To this end, we expressed ApB73‐HA under the constitutive *otef* promoter in the AB33 strain (Brachmann *et al*., 2001; Spellig *et al*., 1996). The genetically modified AB33 strain expresses a heterodimeric transcription factor responsible for filamentation during pathogenic development under a nitrate‐inducible promoter (P_nar_). The ability of AB33 to form filaments after being shifted to nitrate‐containing medium allows for experiments on a developmental stage normally only induced on the plant leaf. In contrast with the *in planta* microscopy data (Fig. 3a), the Western blot performed on the proteins obtained in the secretion assay failed to demonstrate free secretion of ApB73 (Fig. 3b). To narrow down the exact localization of ApB73, a strain was generated that co‐expresses ApB73‐mCherry‐HA and Pit1‐GFP, a known transmembrane protein (Doehlemann *et al*., 2011), each under the native ApB73 promoter. After infection into maize plants, co‐localization of the two fusion proteins was observed (Fig. S5, see Supporting Information). All observations together support the secretion and association of ApB73 to the fungal surface.

The importance of the SP was further analysed by challenging strains expressing different constructs for their ability to complement the *apB73* mutant phenotype in a virulence assay with maize plants. Knowing that the C‐terminal fusion of mCherry to ApB73 fully complements the phenotype (Fig. 3c), the mCherry tag was included in all constructs. This allows the visualization of the fusion protein. The SP was either completely removed (ApB73_20–709_) or substituted by the SP of a previously characterized effector Pit2 (Pit2_1–25_; Doehlemann *et al*., 2011; Fig. 3c). The strain expressing ApB73_20–709_, i.e. lacking the SP, was unable to complement the virulence phenotype. Exchanging the SP from ApB73_1–19_ to Pit2_1–25_ resulted in a strain fully complementing the mutant phenotype. To verify protein expression, microscopy of infected maize leaves was performed (Fig. S6, see Supporting Information). Both constructs showed expression. ApB73_20–709_‐mCherry‐HA was solely localized to the fungal cytoplasm, whereas the Pit2_1–25_ApB73_20–709_‐mCherry‐HA fusion protein was secreted and localized around the hyphae. The complementation assays show not only that the SP is essential for the function of ApB73, but also that it is interchangeable with SP of other proteins.

As a next step, we wanted to exclude the possibility that ApB73, despite being secreted, acts solely in the endoplasmic reticulum (ER). To examine this, we tested whether ApB73, C‐terminally fused to the ER retention signal HDEL, is functional and able to complement the virulence defect of the deletion strain. We introduced ApB73‐mCherry‐HA‐HDEL under the *apB73* promoter ectopically into the *ip* locus of the ΔapB73 strain and performed a virulence assay (Fig. 3c). The resulting strain expressing ApB73‐mCherry‐HA‐HDEL was unable to fully restore virulence; however, small‐ to medium‐sized tumours were formed at a low rate. Microscopic analysis showed that ApB73‐mCherry‐HA‐HDEL is expressed, but not secreted (Fig. S4). Considering that the ApB73‐mCherry‐HA fusion protein complements the virulence phenotype of ΔapB73 and the HDEL sequence is unlikely to cause steric hindrance leading to a non‐functional protein, the fact that some tumours are formed could be explained by a small amount of protein that ‘escapes’ ER retention, quantitatively too low to be observable by microscopy.

Next, we investigated the observation that ApB73 does not seem to be freely secreted to the surrounding medium (Fig. 3b). To do so, we performed microscopy on maize plants infected with a ΔapB73 strain expressing ApB73‐mCherry‐HA under the strong *otef* promoter. Three days later, infected leaf samples were treated with mannitol (1 m) to induce plasmolysis, which allows for the visualization of freely secreted protein, as it should diffuse evenly within the biotrophic interface. This has been demonstrated for other effector fusion proteins, such as Pit2‐mCherry and Pep1‐mCherry (Doehlemann *et al*., 2009, 2011). To exclude the possibility that the ApB73 SP has a membrane‐anchoring function, we fused the SP sequence ApB73_1–19_ to mCherry. The ApB73 SP leads to a clear secretion of mCherry (Fig. 3d, top panel). The mCherry signal is evenly distributed in the apoplast, indicating no membrane or cell wall association of this fusion protein. This means that the ApB73 SP alone is not sufficient to keep a protein associated to the membrane or cell wall. In contrast, if mCherry is fused C‐terminally to full‐length ApB73, no such diffusion can be observed (Fig. 3d, middle panel). The mCherry signal remains exclusively around the hyphae. Interestingly, when performing N‐terminal fusions of mCherry and ApB73, the mCherry signal is detectable in the apoplast (Fig. 3d, bottom panel). This may be an indication that ApB73 is processed before or after secretion into the biotrophic interphase. Another possibility is that the N‐terminally fused mCherry protein sterically hinders the binding of ApB73 to the fungal surface. Both the N‐ and C‐terminal fusions of mCherry to ApB73 fully complement the deletion phenotype (Fig. 3c). As a result, we can conclude that, if mCherry blocks the membrane/cell wall association of ApB73, this association cannot be functionally critical for ApB73.

However, this possible processing seems to be a feature of ApB73 that can only be observed *in planta*. The reasoning here is that, in all protein assays conducted using axenic culture, no size differences were observed and the full‐sized protein was always detected. To this end, we also attempted to conduct immunoprecipitation/Western blot of infected plant material to show the processing of ApB73 biochemically. However, because of the very low expression of ApB73, we could not obtain specific bands. The option of using stronger promoters [e.g. the promoter of *stp1* (Schipper, 2009) or *pep1* (Doehlemann *et al*., 2009); D. Lanver, Max Planck Institute for Terrestrial Microbiology, Marburg, Germany, personal communication] was neglected as the constructed strains could not fully complement the virulence phenotype (Fig. S7, see Supporting Information). The expression and secretion of the fusion proteins were confirmed by microscopy (Fig. S6).

### Orthologues of ApB73 in other smut fungi

**Figure 4:**
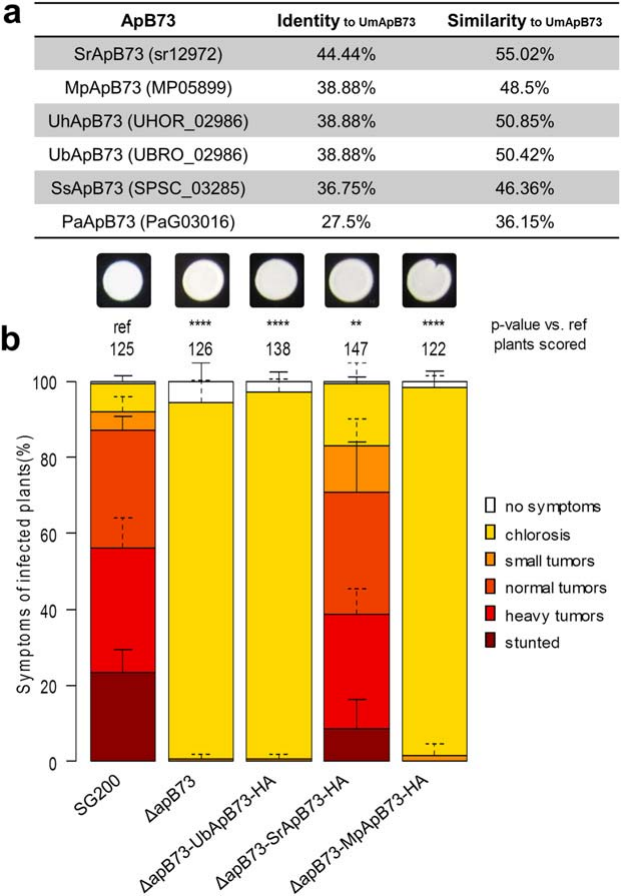
ApB73 has orthologues in other smut fungi. (a) Identity and similarity values of ApB73 orthologues from different smut fungi to the ApB73 protein from *Ustilago maydis*. Included here are protein sequences from *Sporisorium reilianum*, *Melanopsichium pennsylvanicum*, *Ustilago hordei*, *Ustilago bromivora*, *Sporisorium scitamineum* and *Pseudozyma aphidis*. Values were calculated using amino acid sequences and feeding them into the software tool SIAS (Immunomedicine Group, 2015). (b) Disease ratings of different complementation strains of the ΔapB73 mutant. The mutant strain SG200ΔapB73 was transformed with a construct expressing UbApB73, SrApB73 or MpApB73 under the native ApB73 promoter. Seedlings of maize cv. B73 were infected 7 days after germination and scored for symptoms at 12 days post‐infection (dpi). In the top row, photographs of the respective strains are shown after growth on filamentation‐inducing charcoal plates for 24 h. Disease scores are shown on the right. The mean values of three independent infections are depicted and the total number of infected plants is indicated above the respective columns. The mean and standard deviation of relative counts from replicates are displayed. For clarity, only positive error bars are shown. *P* values were calculated by Fisher's exact test. Multiple testing correction was performed using the Benjamini–Hochberg procedure. **P < 0.01; ****P < 0.0001.

Amino acid blast analysis with the ApB73 sequence in related smut genomes revealed the existence of possible ApB73 orthologues in nearly all tested smuts, including the solely saprophytic, apathogenic smut fungus *Pseudozyma aphidis* (Fig. 4a). All orthologues, except the *P. aphidis* protein, share an N‐terminal SP indicating their secretion (Petersen *et al*., 2011). Interestingly, the *Pseudozyma* orthologue PaApB73 also contains a short stretch of amino acids qualifying as an SP. However, because of a new methionine, the potential SP is located 89 amino acids away from the N‐terminus. Identity values among the orthologues range from 27.5% to 44.5%, whereas similarities—a looser definition taking physical and chemical properties of the amino acids into account—reached up to 55% in the *S. reilianum* orthologue (Immunomedicine Group, 2015). We then examined the alignment of the different proteins in order to test for functional conservation of selected ApB73 orthologues (Fig. S8, see Supporting Information). We chose three orthologues for a cross‐species complementation assay: SrApB73 (sr12972) from *S. reilianum*, as the pathogen shares the same host *Z. mays* and is most closely related; MpApB73 (MP05899) from *M. pennsylvanicum*, as it is the only smut infecting dicotyledonous plant; and UbApB73 (UBRO_02986) from *U. bromivora*, a *Bromus*‐infecting smut and close relative of *U. hordei*. As a result of the lack of an exposed SP and the knowledge that a functional SP is essential for ApB73 function, the *Pseudozyma* orthologue was neglected.

For the cross‐species complementation assay, we introduced the coding sequences of the orthologues from *S. reilianum*, *U. bromivora* and *M. pennsylvanicum* into the *U. maydis* ΔapB73 strain by ectopic integration. All orthologues were expressed under the native *apB73* promoter to exclude problems with promoter strength or time of expression. All derived transgenic strains were subjected to a virulence assay in B73 and disease symptoms were scored at 12 days post‐infection (dpi). We found that the *S. reilianum* orthologue SrApB73 was capable of complementing the *apB73* mutant phenotype (Fig. 4b). In contrast, the less conserved orthologues from *U. bromivora* and *M. pennsylvanicum*, both sharing the same identity value towards UmApB73, were unable to rescue the virulence defect of the *apB73* deletion strain (Fig. 4b). To further elaborate whether expression problems could be the reason for UbApB73's inability to complement the *umapB73* deletion phenotype, a C‐terminal mCherry fusion was generated, and expression as well as secretion were confirmed by fluorescence microscopy (Fig. S6).

## Discussion

In recent decades, it has become more and more evident that virulence factors, including effector molecules, are key players in the establishment of biotrophic interactions between pathogens and plants (Jones and Dangl, 2006). Although research has mainly focused on bacterial proteins, factors from oomycetes or fungi have also been characterized, shedding light into these complex interactions (de Jonge *et al*., 2010; Djamei *et al*., 2011).

Here, we describe ApB73, one of the very few known secreted molecules that are crucial to the establishment of biotrophy in the *Ustilago maydis*–*Zea mays* cv. B73 pathosystem. We could show that deletion of *apB73* from the progenitor strain abolishes the ability of the fungus to form tumours on maize cultivar B73. Interestingly, this changed when we employed the most widely used accession in the *U. maydis* field, namely the sweet corn EGB. With this more susceptible maize variety, we commonly observed gall formation when infecting with the ΔapB73 strain, although only to a limited extent. Differences in infection severity in response to different maize accessions have never been described before for any other effector mutant. This observation shows that the outcome of an infection is not only dependent on the strength of the pathogen, but also on the susceptibility of its host.

Additional confirmation comes from the comparison of known quantitative resistance loci. The *U. maydis*–maize interaction is not based on the typical gene‐for‐gene interactions common to other pathosystems. Instead, it was shown that the outcome of an infection of *Z. may*s with *U. maydis* is determined by the interplay of several quantitative trait loci (QTLs; Baumgarten *et al*., 2007). Therefore, although there are currently no studies available comparing the genomes of EGB and B73, we can speculate that differences between these two genomes must affect important quantitative resistance loci decisive for the outcome of an infection with *U. maydis*.

Our results show that virulence phenotypes caused by deletions of ‘weaker’ virulence factors can be intensified when tested in maize accession B73 instead of EGB. Considering how many putative effector gene deletions do not result in a measurable phenotype (Kämper *et al*., 2006), this could help to widen the dynamic range of infection scorings and allow for the detection of minor contributions to virulence from effector candidates. In addition, this could assist researchers in the field of plant pathogens by making them aware that, in some cases, it could help to use various host accessions to elucidate the role of an effector candidate.

From the experiments described here, interesting characteristics with regard to the role of ApB73 can be deduced. In contrast to *pep1* mutants, where the growth of *U. maydis* is blocked at a very early stage, leaving it unable to penetrate the plant (Doehlemann *et al*., 2009), *apB73* mutants seem to be capable of successful penetration of the plant epidermis. However, they fail to establish biotrophy, indicated by the lack of extensive and branched fungal growth as late as 7 dpi. This shows that the expression of *apB73* is crucial for virulence and that the protein is needed, not in the very first steps of the infection process, but later during biotrophic proliferation. The quantitative PCR data shown here support the involvement of ApB73 at later stages of infection. Although the expression of *apB73* is already increased after 24 h (∼10‐fold), highest expression levels are found between 2 and 8 dpi (>100‐fold). This coincides with the biotrophic stages of development of *U. maydis*, as shortly after this phase hyphae start to fragment (9 dpi), initiating the first steps towards the formation of spores (Banuett and Herskowitz, 1996). The expression profile is comparable to that of other known effectors, such as Pit2 or Tin2. Here, deletions also result in strains still capable of penetrating the plant epidermis, but failing to cause symptoms of full virulence. The finding that *apB73* deletion strains are unable to form spores, i.e. progeny, demonstrates that ApB73 is essential for the completion of the sexual, pathogenic life cycle of *U. maydis* inside its host.

The observation that certain effector mutants of *U. maydis* are still virulent to a certain degree, i.e. are, in principle, capable of penetrating the plant and also succeed in colonizing the next few cells, can be explained by imagining an equilibrium between virulence factors and the host defence machinery. The less efficiently the pathogen suppresses the host defence machinery, for example, because of a lack of specific effectors, the more likely it will fail over the long run with regard to the colonization of its host. Vice versa, the less efficiently the host machinery recognizes and reacts to the invader, the more likely it will be overcome by the pathogen. The binary outcome of spore formation is therefore a quantitative product of the susceptibility of the host and the virulence of the pathogen.

*In vivo* microscopy of ApB73‐mCherry and complementation attempts without the ApB73 SP show that ApB73 is secreted and that secretion is essential for its function in virulence. Interestingly, ApB73 seems to stay attached to the fungal membrane or cell wall, as indicated by culture supernatant assays and plasmolysis experiments of infected maize plants using ApB73‐mCherry. *In planta*, we observed free diffusion of ApB73 fusion proteins when conducting plasmolysis experiments with ApB73_1–19_‐mCherry‐3×HA‐ApB73_20–709_3×myc. This either shows a possible processing of ApB73 or steric hindrance of its membrane attachment caused by the N‐terminal mCherry. Processing of this unusually large protein might be an important step that could either change the function of the protein or possibly release an active moiety in order to fulfil its function. Considering the size of the protein, ApB73 may (first) have a shielding function. Selective positioning of ApB73 could make the fungus less obvious to the plant's defence system or shield the fungal membrane against possible defence proteins secreted by the plant. Similar roles are known for virulence factors from other pathogens, including *Cladosporium fulvum* (Avr4; van den Burg *et al*., 2006; van Esse *et al*., 2007) and *Mycosphaerella graminicola* (Mg3LysM; Marshall *et al*., 2011). Avr4 and Mg3LysM protect/shield fungal chitin against hydrolysis by plant chitinases.

Apart from a putative shielding function of ApB73, its possible *in planta* processing may allow other functions. Considering that the processed ApB73 fragments would be much smaller, they may even be translocated into the plant cell.

Also interesting are the observations made when attempting to complement the mutant phenotype using orthologous proteins from closely related smuts. We observed that neither the orthologue from *M. pennsylvanicum* nor that from *U. bromivora* can complement the phenotype. However, the orthologue from *S. reilianum* almost fully restores virulence. This shows that the pure size of the protein is not sufficient for its function, although it should be considered that differences in SP could matter. One reason why only the orthologue from *S. reilianum* can complement the phenotype could be that both fungi are infecting the same host, i.e. both may have evolved to interact with a certain maize protein. A simpler explanation could be the fact that UmApB73 and SrApB73 share the highest identity values. For other effectors, studies of orthologous proteins were also conducted. For Pep1, for example, a well‐characterized effector of *U*. *maydis*, all complementation attempts using orthologues from various other smuts were successful (Hemetsberger *et al*., 2015). The reason here is probably the overall very high conservation (identities of 51%–64% compared with only 27%–44.5% for ApB73). In contrast, complementation attempts for the effector See1 with its *U. hordei* orthologue were not successful, with identity values of 34% (Redkar *et al*., 2015b).

Although we have demonstrated that ApB73 is an important and functionally conserved secreted virulence factor among some of the tested smuts, the elucidation of its mechanistic role during biotrophy is still enigmatic and awaits future research.

## Experimental Procedures

### Strains and plasmids

For all plasmid generations, standard molecular cloning procedures were applied (Sambrook *et al*., 1989). *Escherichia coli* Mach1 (Thermo Fisher Scientific, Waltham, MS, USA) was used for all DNA manipulations. The primers and plasmids generated for this study are shown in Tables S1 and S2 (see Supporting Information). All plasmids used are based on the p123 vector published previously (Aichinger *et al*., 2003). Some plasmids were generated using the GreenGate system (Lampropoulos *et al*., 2013). The modules used were either amplified by PCR (see Table S1 for primer sequences) or DNA was obtained from the published system (Lampropoulos *et al*., 2013). All vector maps containing detailed sequence information are available on request.

*Ustilago maydis* was grown at 28 °C in YEPSL (0.4% yeast extract, 0.4% peptone and 2% sucrose). Pathogenicity assays and disease symptom scoring at 12 dpi were conducted as described previously (Kämper *et al*., 2006). Tassel infections using maize cv. Gaspe Flint were conducted as described previously (Redkar *et al*., 2015a). All *U. maydis* strains (Table S3, see Supporting Information) were generated by gene replacement via homologous recombination with PCR‐generated constructs (Kämper, 2004) or by insertion of p123 derivatives into the *ip* locus, as described previously (Loubradou *et al*., 2001). Isolated *U. maydis* transformants were confirmed by PCR. For the generation of deletion strains, the transformants were additionally tested by southern analysis. For colony growth assays, *U. maydis* strains were spotted onto plates containing complete medium (CM) agar supplemented with 1% glucose and various stress‐inducing compounds in serial dilutions, and incubated for 48 h at 28 °C. The concentrations of these compounds are indicated in the respective experiment. To induce filamentous growth, strains were spotted onto potato dextrose agar containing 1% activated charcoal.

Maize varieties were EGB (Olds Seeds, Madison, WI, USA), Gaspe Flint (kindly provided by Professor Dr Regine Kahmann, Max Planck Institute of Terrestrial Microbiology, Marburg, Germany) or B73 (kindly provided by Professor Dr Alfons Gierl, Wissenschaftszentrum Weihenstephan, Freising‐Weihenstephan, Germany). *Zea mays* was grown in a temperature‐controlled glasshouse (14 h/10 h light/dark cycle; 28 °C/20 °C) and used for infection by *U. maydis*.

### Quantitative real‐time PCR

RNA was extracted from sporidia grown in axenic culture and from infected maize plants at the indicated time points with the TRIzol method (Invitrogen), treated with DNase (Thermo Fisher Scientific, Waltham, MS, USA) and subsequently used for cDNA synthesis. Quantitative real‐time PCRs were conducted as described previously (Doehlemann *et al*., 2008). All reactions were performed in biological triplicates. Relative *apB73* expression levels were calculated in relation to the values obtained for the constitutively expressed peptidyl‐prolyl isomerase gene (*ppi*) of *U. maydis* (Bohlmann, 1996).

### Secretion assay

Secretion assay was performed as described by Djamei *et al*. (2011). In brief, after filament induction, the cell‐free supernatant was precipitated with trichloroacetic acid (TCA)/Sodium deoxycholate (DOC) and a Western blot was performed on the precipitate. *Ustilago maydis* strain AB33 P_otef_apB73‐HA was generated by insertion of plasmid p123‐P_otef_‐ApB73‐HA into the *ip* locus of AB33 (Brachmann *et al*., 2001). AB33 cell extracts and supernatants were used to ensure specificity of the antibodies. Actin detection served as a lysis control and bands could only be observed for whole cell extracts. For Western blot analysis, mouse anti‐haemagglutinin (anti‐HA) (Sigma‐Aldrich, St. Louis, MO, USA) and mouse anti‐actin (Invitrogen, Waltham, MA, USA) antibodies were used. Assays were carried out at least three times.

### Microscopy

To visualize fungal proliferation in infected tissue, the area 1–3 cm below the injection site was excised at 2–7 dpi. Fungal hyphae, i.e. chitin, were stained with wheatgerm agglutinin (WGA) coupled to AlexaFluor488 (Invitrogen). Plant cell walls were stained with propidium iodide (Sigma‐Aldrich, St. Louis, MO, USA). Leaf samples were incubated in staining solution (1 µg/mL propidium iodide, 10 µg/mL WGA‐AF488) and observed with an LSM780 Axio Observer confocal laser scanning microscope (Zeiss, Jena, Germany) under the following conditions: WGA‐AF488, excitation at 488 nm and detection at 500–540 nm; propidium iodide, excitation at 561 nm and detection at 580–660 nm. For fluorescent protein detection, leaf samples were directly observed by confocal microscopy using the following conditions for mCherry: excitation at 561 nm and detection at 580–630 nm. For plasmolysis experiments, a 1 m mannitol solution was infiltrated into the leaves and observed 30 min later. All experiments were carried out at least three times. To measure appressoria formation and penetration efficiency, infected maize leaves were harvested 18–20 h post‐infection and stained with calcofluor white (10 ng/mL). Calcofluor white stains fungal hyphae only on the leaf surface as it cannot stain penetrated hyphae. The appressorial marker used has been described previously (Mendoza‐Mendoza *et al*., 2009).

### Bioinformatic analysis

Gene and protein sequences of *U. maydis*, *S. reilianum* and *U. hordei* were taken from MIPS Ustilago Maydis Database/MIPS Ustilago Hordei Database (MUMDB/MUHDB) and MIPS Sporisorium Reilianum Database (MSRDB) Munich Information Center for Protein Sequences (MIPS), respectively (https://www.helmholtz-muenchen.de/en/ibis/institute/groups/fungal‐microbial‐genomics/resources/ustilaginaceae/index.html). Sequences of *M. pennsylvanicum* and *P. aphidis* were taken from the National Center for Biotechnology Information (NCBI). *Ustilago bromivora* sequences were identified by Sanger sequencing of PCR products obtained with degenerated *U. hordei* primers on *U. bromivora* genomic DNA. Homology analyses were performed using blast (Basic Local Alignment Search Tool). Homologous amino acid sequences were compared using CLC Main Workbench 7 (Qiagen, Hilden, Germany). SP prediction was performed with the program SignalP4.1 (http://www.cbs.dtu.dk/services/SignalP/).

For disease scoring evaluation, an R‐script was used processing the data as described below. Class counts for each genotype of the three biological replicates were summarized. Then, for each pair of genotypes, Fisher's exact test was used to evaluate whether the distribution of counts between classes was significantly different between the genotypes. The resulting *P* values were multiple testing corrected by the Benjamini–Hochberg algorithm (Benjamini and Hochberg, 1995). For figures, the counts for each genotype and replicate were converted to relative counts. Error bars depict the standard deviation of the relative counts in the three replicates. The significance of each genotype in comparison with the defined reference is indicated by asterisks.

## Supporting information

Additional Supporting Information may be found in the online version of this article at the publisher's website:

**Fig. S1** Filament induction on charcoal plates after mating wild‐type strains FB1 and FB2. *Ustilago maydis* pre‐cultures of the two wild‐type strains FB1 and FB2 were grown to an optical density at 600 nm (OD_600_) of 1.0 and individually or as a mixture dropped onto charcoal plates. The two strains, FB1 and FB2, have compatible mating types that allow them to mate (recognizable by the fuzzy/whiter colony morphology), whereas strains with the same mating type cannot mate when mixed together, resulting in no filament formation. The photograph was taken 24 h after dropping. Bar, 0.5 cm.
**Fig. S2** Effect of ApB73 on growth under stress conditions. Growth of *Ustilago maydis* SG200, SG200ΔapB73 and SG200ΔapB73‐P_oma_‐apB73 on media providing different stresses. First, *U. maydis* pre‐cultures were grown to an optical density at 600 nm (OD_600_) of 1.0. Cells were washed in water and 10‐fold serial dilutions were prepared. From each solution, 7 µL were dropped onto the different media. Photographs were taken 48 h later. Media used from top left to bottom right: complete medium (CM) agar containing 1 m NaCl; nitrate minimal medium; CM agar supplied with Congo red (70 µg/mL); CM agar supplied with calcofluor (45 µg/mL); ammonium minimal medium; CM agar supplied with 1.5 mm H_2_O_2_.
**Fig. S3** Appressorial marker induction and penetration efficiency of the *apB73* mutant compared with SG200. (a) Representative photograph showing the analysed filaments on an infected maize leaf. For infection, two strains (SG200AM1‐RFP and SG200ΔapB73‐AM1) at an optical density at 600 nm (OD_600_) of unity were mixed, infected into maize cv. B73 and stained with calcofluor white at 18–20 h post‐infection. SG200AM1 cells appear red (red fluorescent protein, RFP) and blue (calcofluor white stain). SG200AM1 cells, after induction of the appressorial marker, appear blue/red and green (green fluorescent protein, GFP). SG200ΔapB73‐AM1 strains appear blue or green/blue after induction of the appressorial marker. Bar, 10 µm. (b) Comparison of the induced appressorial marker in SG200AM1‐RFP or SG200ΔapB73‐AM1. Three independent plants were analysed, as efficiency varies greatly depending on the analysed area and plant. The experiment was repeated twice with similar results. (c) Penetration efficiency of the appressoria formed in SG200AM1‐RFP or SG200ΔapB73‐AM1. Penetrated hyphae expressing cytoplasmic GFP cannot be stained by calcofluor white, allowing the quantification of the penetration efficiency (ratio of green vs. blue/green hyphae).
**Fig. S4  **Spore formation is impaired after infection with FB1ΔapB73×FB2ΔapB73 on maize cv. Gaspe Flint. (a) Tassel phenotype of maize plants cv. Gaspe Flint infected with either FB1×FB2 or FB1ΔapB73×FB2ΔapB73. Representative tassels were chosen to illustrate spore development. The photograph was taken at 18 days post‐infection (dpi). White arrows indicate black teliospores. Bar, 1 cm. (b–d) Microscopic analysis of infected tassels labelled with WGA AlexaFluor488 at 18 dpi. Bar, 20 µm. (b) Germinating spores observed in tassels infected with FB1×FB2. (c) Tumour tissue without detectable *Ustilago maydis* cells observed on tassels of plants infected with FB1ΔapB73×FB2ΔapB73. (d) Fungal hyphae of *U. maydis* on tassels infected with FB1ΔapB73×FB2ΔapB73.
**Fig. S5** Localization of Pit1‐GFP and ApB73‐mCherry‐HA in infected maize leaves. Seven‐day‐old maize seedlings of cv. B73 were infected with a ΔapB73 strain co‐expressing ApB73‐mCherry‐HA and Pit1‐GFP under the native ApB73 promoter. Fluorescence was observed by confocal microscopy at 3 days post‐infection. Bar, 5 μm.
**Fig. S6** Microscopy of infected maize leaves showing the expression and localization of the fusion proteins used. Seven‐day‐old maize seedlings of cv. B73 were infected with ΔapB73 strains expressing ApB73_20–709_‐mCherryHA, Pit2_1–25_‐ApB73_20–709_‐mCherryHA, ApB73‐mCherry‐HDEL or ApB73_1–19_‐mCherry‐3×HA‐ApB73_20–709_−3×myc under the native ApB73 promoter. In addition, maize tissues infected with ΔapB73 strains expressing ApB73‐mCherry‐HA under either the *pit2* or *stp1* promoter are shown. Fluorescence was observed at 5 days post‐infection. Bar, 10 μm.
**Fig. S7** Virulence assay using promoters strongly up‐regulated during biotrophy. Disease rating of ApB73 expressed under different promoters in the ΔapB73 mutant. The mutant was transformed with a construct expressing ApB73‐mCherry‐HA under two biotrophy‐specific promoters. The promoters used express the effector genes *stp1* and *pit2* (Doehlemann *et al*., 2011; Schipper, 2009). Seedlings of maize cv. B73 were infected 7 days after germination and scored for symptoms at 12 days post‐infection (dpi). In the top row, photographs of the respective strains are shown after growth on filamentation‐inducing charcoal plates for 24 h. Disease scores are shown on the right. The mean values of three independent infections are depicted and the total number of infected plants is indicated above the respective columns. The mean and standard deviation of relative counts from replicates are displayed. For clarity, only positive error bars are shown. *P* values were calculated by Fisher's exact test. Multiple testing correction was performed using the Benjamini–Hochberg procedure. ****P < 0.0001.
**Fig. S8  **Amino acid sequence alignment of ApB73 orthologues from different smuts. Alignment sequences of the full‐length proteins from *Ustilago maydis* (UmApB73), *Ustilago bromivora* (UbApB73), *Ustilago hordei* (UhApB73), *Sporisorium reilianum* (SrApB73), *Sporisorium scitamineum* (ScApB73), *Melanopsichium pennsylvanicum* (MpApB73) and *Pseudozyma aphidis* (PaGApB73) were obtained from public databases. Sequence alignment was generated by the multiple sequence alignment program clustal O in CLC main bench using default parameters. The colour scheme shows the consensus strength, with red representing the highest and blue the lowest values.

**Table S1** Primers used in this study.
**Table S2** Plasmids used in this study.
**Table S3** *Ustilago maydis* strains used in this study.

## References

[mpp12442-bib-0001] Agrios, G.N. (1988) Plant Pathology. New York: Academic Press.

[mpp12442-bib-0002] Aichinger, C. , Hansson, K. , Eichhorn, H. , Lessing, F. , Mannhaupt, G. , Mewes, W. and Kahmann, R. (2003) Identification of plant‐regulated genes in *Ustilago maydis* by enhancer‐trapping mutagenesis. Mol. Genet. Genomics, 270, 303–314. 14523645 10.1007/s00438-003-0926-z

[mpp12442-bib-0003] Banuett, F. and Herskowitz, I. (1996) Discrete developmental stages during teliospore formation in the corn smut fungus, *Ustilago maydis* . Development, 122, 2965–2976. 8898211 10.1242/dev.122.10.2965

[mpp12442-bib-0004] Baumgarten, A.M. , Suresh, J. , May, G. and Phillips, R.L. (2007) Mapping QTLs contributing to *Ustilago maydis* resistance in specific plant tissues of maize. Theor. Appl. Genet. 114, 1229–1238. 17468806 10.1007/s00122-007-0513-5

[mpp12442-bib-0005] Benjamini, Y. and Hochberg, Y. (1995) Controlling the false discovery rate: a practical and powerful approach to multiple testing. J. R. Stat. Soc. 57, 289–300.

[mpp12442-bib-0006] Bohlmann, R. (1996) Isolierung und Charakterisierung von filamentspezifisch experimierten Genen aus *Ustilago maydis*. Munich: Ludwig‐Maximilian‐Universität .

[mpp12442-bib-0007] Brachmann, A. , Weinzierl, G. , Kamper, J. and Kahmann, R. (2001) Identification of genes in the bW/bE regulatory cascade in *Ustilago maydis* . Mol. Microbiol. 42, 1047–1063. 11737646 10.1046/j.1365-2958.2001.02699.x

[mpp12442-bib-0008] Brefort, T. , Doehlemann, G. , Mendoza‐Mendoza, A. , Reissmann, S. , Djamei, A. and Kahmann, R. (2009) *Ustilago maydis* as a pathogen. Annu. Rev. Phytopathol. 47, 423–445. 19400641 10.1146/annurev-phyto-080508-081923

[mpp12442-bib-0009] van den Burg, H.A. , Harrison, S.J. , Joosten, M.H. , Vervoort, J. and de Wit, P.J. (2006) *Cladosporium fulvum* Avr4 protects fungal cell walls against hydrolysis by plant chitinases accumulating during infection. Mol. Plant–Microbe Interact. 19, 1420–1430. 17153926 10.1094/MPMI-19-1420

[mpp12442-bib-0010] Buxdorf, K. , Rahat, I. , Gafni, A. and Levy, M. (2013) The epiphytic fungus *Pseudozyma aphidis* induces jasmonic acid‐ and salicylic acid/nonexpressor of PR1‐independent local and systemic resistance. Plant Physiol. 161, 2014–2022. 23388119 10.1104/pp.112.212969 PMC3613472

[mpp12442-bib-0011] Christensen, J.J. (1963) Corn smut caused by *Ustilago maydis* . Am. Phytopathol. Soc. Monogr. 2, 35–41.

[mpp12442-bib-0012] de Jonge, R. , van Esse, H.P. , Kombrink, A. , Shinya, T. , Desaki, Y. , Bours, R. , van der Krol, S. , Shibuya, N. , Joosten, M.H.A.J. and Thomma, B.P.H.J. (2010) Conserved fungal LysM effector Ecp6 prevents chitin‐triggered immunity in plants. Science, 329, 953–955. 20724636 10.1126/science.1190859

[mpp12442-bib-0013] de Jonge, R. , Bolton, M.D. and Thomma, B.P.H.J. (2011) How filamentous pathogens co‐opt plants: the ins and outs of fungal effectors. Curr. Opin. Plant Biol. 14, 400–406. 21454120 10.1016/j.pbi.2011.03.005

[mpp12442-bib-0014] Djamei, A. and Kahmann, R. (2012) *Ustilago maydis*: dissecting the molecular interface between pathogen and plant. PLOS Pathog. 8, e1002955. 10.1371/journal.ppat.1002955 PMC3486881 23133380

[mpp12442-bib-0015] Djamei, A. , Schipper, K. , Rabe, F. , Ghosh, A. , Vincon, V. , Kahnt, J. , Osorio, S. , Tohge, T. , Fernie, A.R. , Feussner, I. , Meinicke, P. , Stierhof, Y.D. , Schwarz, H. , Macek, B. , Mann, M. and Kahmann, R. (2011) Metabolic priming by a secreted fungal effector. Nature, 478, 395–398. 21976020 10.1038/nature10454

[mpp12442-bib-0016] Doehlemann, G. , Wahl, R. , Horst, R.J. , Voll, L.M. , Usadel, B. , Poree, F. , Stitt, M. , Pons‐Kühnemann, J. , Sonnewald, U. , Kahmann, R. and Kämper, J. (2008) Reprogramming a maize plant: transcriptional and metabolic changes induced by the fungal biotroph *Ustilago maydis* . Plant J. 56, 181–195. 18564380 10.1111/j.1365-313X.2008.03590.x

[mpp12442-bib-0017] Doehlemann, G. , van der Linde, K. , Aßmann, D. , Schwammbach, D. , Hof, A. , Mohanty, A. , Jackson, D. and Kahmann, R. (2009) Pep1, a secreted effector protein of *Ustilago maydis*, is required for successful invasion of plant cells. PLOS Pathog. 5, e1000290. 19197359 10.1371/journal.ppat.1000290 PMC2631132

[mpp12442-bib-0018] Doehlemann, G. , Reissmann, S. , Aßmann, D. , Fleckenstein, M. and Kahmann, R. (2011) Two linked genes encoding a secreted effector and a membrane protein are essential for *Ustilago maydis*‐induced tumour formation. Mol. Microbiol. 81, 751–766. 21692877 10.1111/j.1365-2958.2011.07728.x

[mpp12442-bib-0019] Dolgin, E. (2009) Maize genome mapped. Nature. doi:10.1038/news.2009.1098.

[mpp12442-bib-0020] van Esse, H.P. , Bolton, M.D. , Stergiopoulos, I. , de Wit, P.J.G.M. and Thomma, B.P.H.J. (2007) The chitin‐binding *Cladosporium fulvum* effector protein Avr4 is a virulence factor. Mol. Plant–Microbe Interact. 20, 1092–1101. 17849712 10.1094/MPMI-20-9-1092

[mpp12442-bib-0021] Flor‐Parra, I. (2006) Biz1, a zinc finger protein required for plant invasion by *Ustilago maydis*, regulates the levels of a mitotic cyclin. Plant Cell, 18, 2369–2387. 16905655 10.1105/tpc.106.042754 PMC1560913

[mpp12442-bib-0022] Hemetsberger, C. , Herrberger, C. , Zechmann, B. , Hillmer, M. and Doehlemann, G. (2012) The *Ustilago maydis* effector Pep1 suppresses plant immunity by inhibition of host peroxidase activity. PLOS Pathog. 8, e1002684. 22589719 10.1371/journal.ppat.1002684 PMC3349748

[mpp12442-bib-0023] Hemetsberger, C. , Mueller, A.N. , Matei, A. , Herrberger, C. , Hensel, G. , Kumlehn, J. , Mishra, B. , Sharma, R. , Thines, M. , Hückelhoven, R. and Doehlemann, G. (2015) The fungal core effector Pep1 is conserved across smuts of dicots and monocots. New Phytol. 206, 1116–1126. 25628012 10.1111/nph.13304

[mpp12442-bib-0024] Immunomedicine Group UCdM (2015) SIAS – Software to Calculate Sequence Identity and Similarity. Available at. http://imed.med.ucm.es/Tools/sias.html [accessed 21 February 2016].

[mpp12442-bib-0025] Jia, Y. , McAdams, S.A. , Bryan, G.T. , Hershey, H.P. and Valent, B. (2000) Direct interaction of resistance gene and avirulence gene products confers rice blast resistance. EMBO J. 19, 4004–4014. 10921881 10.1093/emboj/19.15.4004 PMC306585

[mpp12442-bib-0026] Jones, J.D.G. and Dangl, J.L. (2006) The plant immune system. Nature, 444, 323–329. 17108957 10.1038/nature05286

[mpp12442-bib-0027] Kahmann, R. and Kämper, J. (2004) *Ustilago maydis*: how its biology relates to pathogenic development. New Phytol. 164, 31–42. 10.1111/j.1469-8137.2004.01156.x 33873482

[mpp12442-bib-0028] Kämper, J. (2004) A PCR‐based system for highly efficient generation of gene replacement mutants in *Ustilago maydis* . Mol. Genet. Genomics, 271, 103–110. 14673645 10.1007/s00438-003-0962-8

[mpp12442-bib-0029] Kämper, J. , Kahmann, R. , Bölker, M. , Ma, L.‐J. , Brefort, T. , Saville, B.J. , Banuett, F. , Kronstad, J.W. , Gold, S.E. , Müller, O. , Perlin, M.H. , Wösten, H.A. , de Vries, R. , Ruiz‐Herrera, J. , Reynaga‐Peña, C.G. , Snetselaar, K. , McCann, M. , Pérez‐Martín, J. , Feldbrügge, M. , Basse, C.W. , Steinberg, G. , Ibeas, J.I. , Holloman, W. , Guzman, P. , Farman, M. , Stajich, J.E. , Sentandreu, R. , González‐Prieto, J.M. , Kennell, J.C. , Molina, L. , Schirawski, J. , Mendoza‐Mendoza, A. , Greilinger, D. , Münch, K. , Rössel, N. , Scherer, M. , Vranes, M. , Ladendorf, O. , Vincon, V. , Fuchs, U. , Sandrock, B. , Meng, S. , Ho, E.C. , Cahill, M.J. , Boyce, K.J. , Klose, J. , Klosterman, S.J. , Deelstra, H.J. , Ortiz‐Castellanos, L. , Li, W. , Sanchez‐Alonso, P. , Schreier, P.H. , Häuser‐Hahn, I. , Vaupel, M. , Koopmann, E. , Friedrich, G. , Voss, H. , Schlüter, T. , Margolis, J. , Platt, D. , Swimmer, C. , Gnirke, A. , Chen, F. , Vysotskaia, V. , Mannhaupt, G. , Güldener, U. , Münsterkötter, M. , Haase, D. , Oesterheld, M. , Mewes, H.W. , Mauceli, E.W. , DeCaprio, D. , Wade, C.M. , Butler, J. , Young, S. , Jaffe, D.B. , Calvo, S. , Nusbaum, C. , Galagan, J. and Birren, B.W. (2006) Insights from the genome of the biotrophic fungal plant pathogen *Ustilago maydis* . Nature, 444, 97–101. 17080091 10.1038/nature05248

[mpp12442-bib-0030] Keon, J.P.R. , White, G.A. and Hargreaves, J.A. (1991) Isolation, characterization and sequence of a gene conferring resistance to the systemic fungicide carboxin from the maize smut pathogen, *Ustilago maydis* . Curr. Genet. 19, 475–481. 1879000 10.1007/BF00312739

[mpp12442-bib-0031] Koeck, M. , Hardham, A.R. and Dodds, P.N. (2011) The role of effectors of biotrophic and hemibiotrophic fungi in infection. Cell. Microbiol. 13, 1849–1857. 21848815 10.1111/j.1462-5822.2011.01665.x PMC3218205

[mpp12442-bib-0032] Lampropoulos, A. , Sutikovic, Z. , Wenzl, C. , Maegele, I. , Lohmann, J.U. and Forner, J. (2013) GreenGate – a novel, versatile, and efficient cloning system for plant transgenesis. PLoS One, 8, e83043. 24376629 10.1371/journal.pone.0083043 PMC3869738

[mpp12442-bib-0033] Le Roux, C. , Huet, G. , Jauneau, A. , Camborde, L. , Trémousaygue, D. , Kraut, A. , Zhou, B. , Levaillant, M. , Adachi, H. , Yoshioka, H. , Raffaele, S. , Berthomé, R. , Couté, Y. , Parker, J.E. and Deslandes, L. (2015) A receptor pair with an integrated decoy converts pathogen disabling of transcription factors to immunity. Cell, 161, 1074–1088. 26000483 10.1016/j.cell.2015.04.025

[mpp12442-bib-0034] Lindeberg, M. , Cunnac, S. and Collmer, A. (2012) *Pseudomonas syringae* type III effector repertoires: last words in endless arguments. Trends Microbiol. 20, 199–208. 22341410 10.1016/j.tim.2012.01.003

[mpp12442-bib-0035] Loubradou, G. , Brachmann, A. , Feldbrugge, M. and Kahmann, R. (2001) A homologue of the transcriptional repressor Ssn6p antagonizes cAMP signalling in *Ustilago maydis* . Mol. Microbiol. 40, 719–730. 11359577 10.1046/j.1365-2958.2001.02424.x

[mpp12442-bib-0036] Marshall, R. , Kombrink, A. , Motteram, J. , Loza‐Reyes, E. , Lucas, J. , Hammond‐Kosack, K.E. , Thomma, B.P.H.J. and Rudd, J.J. (2011) Analysis of two in planta expressed LysM effector homologs from the fungus *Mycosphaerella graminicola* reveals novel functional properties and varying contributions to virulence on wheat. Plant Physiol. 156, 756–769. 21467214 10.1104/pp.111.176347 PMC3177273

[mpp12442-bib-0037] Mendoza‐Mendoza, A. , Berndt, P. , Djamei, A. , Weise, C. , Linne, U. , Marahiel, M. , Vraneš, M. , Kämper, J. and Kahmann, R. (2009) Physical‐chemical plant‐derived signals induce differentiation in *Ustilago maydis* . Mol. Microbiol. 71, 895–911. 19170880 10.1111/j.1365-2958.2008.06567.x

[mpp12442-bib-0038] Mentlak, T.A. , Kombrink, A. , Shinya, T. , Ryder, L.S. , Otomo, I. , Saitoh, H. , Terauchi, R. , Nishizawa, Y. , Shibuya, N. , Thomma, B.P.H.J. and Talbot NJ. (2012) Effector‐mediated suppression of chitin‐triggered immunity by *Magnaporthe oryzae* is necessary for rice blast disease. Plant Cell, 24, 322–335. 22267486 10.1105/tpc.111.092957 PMC3289562

[mpp12442-bib-0039] Mueller, A.N. , Ziemann, S. , Treitschke, S. , Aßmann, D. and Doehlemann, G. (2013) Compatibility in the *Ustilago maydis*–maize interaction requires inhibition of host cysteine proteases by the fungal effector Pit2. PLOS Pathog. 9, e1003177. 23459172 10.1371/journal.ppat.1003177 PMC3573112

[mpp12442-bib-0040] Mukhtar, M.S. , Carvunis, A.R. , Dreze, M. , Epple, P. , Steinbrenner, J. , Moore, J. , Tasan, M. , Galli, M. , Hao, T. , Nishimura, M.T. , Pevzner, S.J. , Donovan, S.E. , Ghamsari, L. , Santhanam, B. , Romero, V. , Poulin, M.M. , Gebreab, F. , Gutierrez, B.J. , Tam, S. , Monachello, D. , Boxem, M. , Harbort, C.J. , McDonald, N. , Gai, L. , Chen, H. , He, Y. , European Union Effectoromics Consortium , Vandenhaute, J. , Roth, F.P. , Hill, D.E. , Ecker, J.R. , Vidal, M. , Beynon, J. , Braun, P. and Dangl, J.L. (2011) Independently evolved virulence effectors converge onto hubs in a plant immune system network. Science, 333, 596–601. 21798943 10.1126/science.1203659 PMC3170753

[mpp12442-bib-0041] Petersen, T.N. , Brunak, S. , von Heijne, G. and Nielsen, H. (2011) SignalP 4.0: discriminating signal peptides from transmembrane regions. Nat. Methods, 8, 785–786. 21959131 10.1038/nmeth.1701

[mpp12442-bib-0042] Redkar, A. , Hoser, R. , Schilling, L. , Zechmann, B. , Krzymowska, M. , Walbot, V. and Doehlemann, G. (2015a) A secreted effector protein of *Ustilago maydis* guides maize leaf cells to form tumors. Plant Cell, 27, 1332–1351. 25888589 10.1105/tpc.114.131086 PMC4558682

[mpp12442-bib-0043] Redkar, A. , Villajuana‐ Bonequi, M. and Doehlemann, G. (2015b) Conservation of the *Ustilago maydis* effector See1 in related smuts. Plant Signal. Behav. 10, e1086855. 26357869 10.1080/15592324.2015.1086855 PMC4854346

[mpp12442-bib-0044] Saitoh, H. , Fujisawa, S. , Mitsuoka, C. , Ito, A. , Hirabuchi, A. , Ikeda, K. , Irieda, H. , Yoshino, K. , Yoshida, K. , Matsumura, H. , Tosa, Y. , Win, J. , Kamoun, S. , Takano, Y. and Terauchi, R. (2012) Large‐scale gene disruption in *Magnaporthe oryzae* identifies MC69, a secreted protein required for infection by monocot and dicot fungal pathogens. PLOS Pathog. 8, e1002711. 22589729 10.1371/journal.ppat.1002711 PMC3349759

[mpp12442-bib-0045] Sambrook, J. , Fritsch, E.F. and Maniatis, T. (1989) Molecular Cloning: A Laboratory Manual. New York: Cold Spring Harbor Laboratory Press.

[mpp12442-bib-0046] Schilling, L. , Matei, A. , Redkar, A. , Walbot, V. and Doehlemann, G. (2014) Virulence of the maize smut *Ustilago maydis* is shaped by organ‐specific effectors. Mol. Plant Pathol. 15, 780–789. 25346968 10.1111/mpp.12133 PMC6638905

[mpp12442-bib-0047] Schipper, K. (2009) Charakterisierung Eines Ustilago Maydis Genclusters, Das Für Drei Neuartige Sekretierte Effektoren Kodiert. Marburg: Philipps‐Universität.

[mpp12442-bib-0048] Schirawski, J. , Mannhaupt, G. , Munch, K. , Brefort, T. , Schipper, K. , Doehlemann, G. , Di Stasio, M. , Rossel, N. , Mendoza‐Mendoza, A. , Pester, D. , Müller, O. , Winterberg, B. , Meyer, E. , Ghareeb, H. , Wollenberg, T. , Münsterkötter, M. , Wong, P. , Walter, M. , Stukenbrock, E. , Güldener, U. and Kahmann, R. (2010) Pathogenicity determinants in smut fungi revealed by genome comparison. Science, 330, 1546–1548. 21148393 10.1126/science.1195330

[mpp12442-bib-0049] Schnable, P.S. , Ware, D. , Fulton, R.S. , Stein, J.C. , Wei, F.S. , Pasternak, S. , Liang, C.Z. , Zhang, J.W. , Fulton, L. , Graves, T.A. , Minx, P. , Reily, A.D. , Courtney, L. , Kruchowski, S.S. , Tomlinson, C. , Strong, C. , Delehaunty, K. , Fronick, C. , Courtney, B. , Rock, S.M. , Belter, E. , Du, F. , Kim, K. , Abbott, R.M. , Cotton, M. , Levy, A. , Marchetto, P. , Ochoa, K. , Jackson, S.M. , Gillam, B. , Chen, W. , Yan, L. , Higginbotham, J. , Cardenas, M. , Waligorski, J. , Applebaum, E. , Phelps, L. , Falcone, J. , Kanchi, K. , Thane, T. , Scimone, A. , Thane, N. , Henke, J. , Wang, T. , Ruppert, J. , Shah, N. , Rotter, K. , Hodges, J. , Ingenthron, E. , Cordes, M. , Kohlberg, S. , Sgro, J. , Delgado, B. , Mead, K. , Chinwalla, A. , Leonard, S. , Crouse, K. , Collura, K. , Kudrna, D. , Currie, J. , He, R. , Angelova, A. , Rajasekar, S. , Mueller, T. , Lomeli, R. , Scara, G. , Ko, A. , Delaney, K. , Wissotski, M. , Lopez, G. , Campos, D. , Braidotti, M. , Ashley, E. , Golser, W. , Kim, H. , Lee, S. , Lin, J. , Dujmic, Z. , Kim, W. , Talag, J. , Zuccolo, A. , Fan, C. , Sebastian, A. , Kramer, M. , Spiegel, L. , Nascimento, L. , Zutavern, T. , Miller, B. , Ambroise, C. , Muller, S. , Spooner, W. , Narechania, A. , Ren, L. , Wei, S. , Kumari, S. , Faga, B. , Levy, M.J. , McMahan, L. , Van Buren, P., Vaughn, M.W. , Ying, K. , Yeh, C.T. , Emrich, S.J., Jia, Y. , Kalyanaraman, A. , Hsia, A.P. , Barbazuk, W.B., Baucom, R.S. , Brutnell, T.P., Carpita, N.C. , Chaparro, C. , Chia, J.M. , Deragon, J.M., Estill, J.C. , Fu, Y. , Jeddeloh, J.A. , Han, Y. , Lee, H. , Li, P. , Lisch, D.R. , Liu, S. , Liu, Z. , Nagel, D.H. , McCann, M.C., SanMiguel, P. , Myers, A.M. , Nettleton, D. , Nguyen, J. , Penning, B.W. , Ponnala, L. , Schneider, K.L. , Schwartz, D.C., Sharma, A. , Soderlund, C. , Springer, N.M. , Sun, Q. , Wang, H. , Waterman, M. , Westerman, R. , Wolfgruber, T.K. , Yang, L. , Yu, Y. , Zhang, L. , Zhou, S. , Zhu, Q. , Bennetzen, J.L. , Dawe, R.K., Jiang, J. , Jiang, N. , Presting, G.G. , Wessler, S.R., Aluru, S. , Martienssen, R.A. , Clifton, S.W., McCombie, W.R. , Wing, R.A. and Wilson, R.K. (2009) The B73 maize genome: complexity, diversity, and dynamics. Science, 326, 1112–1115. 19965430 10.1126/science.1178534

[mpp12442-bib-0050] Sharma, R. , Mishra, B. , Runge, F. and Thines, M. (2014) Gene loss rather than gene gain is associated with a host jump from monocots to dicots in the smut fungus *Melanopsichium pennsylvanicum* . Genome Biol. Evol. 6, 2034–2049. 25062916 10.1093/gbe/evu148 PMC4159001

[mpp12442-bib-0051] Skibbe, D.S. , Doehlemann, G. , Fernandes, J. and Walbot, V. (2010) Maize tumors caused by *Ustilago maydis* require organ‐specific genes in host and pathogen. Science, 328, 89–92. 10.1126/science.1185775 20360107

[mpp12442-bib-0052] Spellig, T. , Bottin, A. and Kahmann, R. (1996) Green fluorescent protein (GFP) as a new vital marker in the phytopathogenic fungus *Ustilago maydis* . Mol. Gen. Genet. 252, 503–509. 8914511 10.1007/BF02172396

[mpp12442-bib-0053] Sperschneider, J. , Dodds, P.N. , Gardiner, D.M. , Manners, J.M. , Singh, K.B. and Taylor, J.M. (2015) Advances and challenges in computational prediction of effectors from plant pathogenic fungi. PLoS Pathog. 11, e1004806. 26020524 10.1371/journal.ppat.1004806 PMC4447458

[mpp12442-bib-0054] Stoll, M. , Piepenbring, M. , Begerow, D. and Oberwinkler, F. (2003) Molecular phylogeny of *Ustilago* and *Sporisorium* species (Basidiomycota, Ustilaginales) based on internal transcribed spacer (ITS) sequences. Can. J. Bot. 81, 976–984.

[mpp12442-bib-0055] Tanaka, S. , Brefort, T. , Neidig, N. , Djamei, A. , Kahnt, J. , Vermerris, W. , Koenig, S. , Feussner, K. , Feussner, I. and Kahmann, R. (2014) A secreted *Ustilago maydis* effector promotes virulence by targeting anthocyanin biosynthesis in maize. eLife, 3, e01355. 10.7554/eLife.01355 PMC3904489 24473076

[mpp12442-bib-0056] White, D.G. (1999) Compendium of Corn Diseases. St. Paul, MN: APS Press.

[mpp12442-bib-0057] Wisser, R.J. , Balint‐Kurti, P.J. and Nelson, R.J. (2006) The genetic architecture of disease resistance in maize: a synthesis of published studies. Phytopathology, 96, 120–129. 18943914 10.1094/PHYTO-96-0120

