# Supplementary material for: Characterization of ApB73, a virulence factor important for colonization of Zea mays by the smut Ustilago maydis
Source: Mol Plant Pathol. 2016 Aug 8;17(9):1467–79. doi: 10.1111/mpp.12442 (PMC5132131; doi:10.1111/mpp.12442)
Supplement: Supplementary file 1 — Fig. S1 Filament induction on charcoal plates after mating wild‐type strains FB1 and FB2. Ustilago maydis pre‐cultures of the two wild‐type strains FB1 and FB2 were grown to an optical density at 600 nm (OD600) of 1.0 and individually or as a mixture dropped onto charcoal plates. The two strains, FB1 and FB2, have compatible mating types that allow them to mate (recognizable by the fuzzy/whiter colony morphology), whereas strains with the same mating type cannot mate when mixed together, resulting in no filament formation. The photograph was taken 24 h after dropping. Bar, 0.5 cm. Fig. S2 Effect of ApB73 on growth under stress conditions. Growth of Ustilago maydis SG200, SG200ΔapB73 and SG200ΔapB73‐Poma‐apB73 on media providing different stresses. First, U. maydis pre‐cultures were grown to an optical density at 600 nm (OD600) of 1.0. Cells were washed in water and 10‐fold serial dilutions were prepared. From each solution, 7 µL were dropped onto the different media. Photographs were taken 48 h later. Media used from top left to bottom right: complete medium (CM) agar containing 1 m NaCl; nitrate minimal medium; CM agar supplied with Congo red (70 µg/mL); CM agar supplied with calcofluor (45 µg/mL); ammonium minimal medium; CM agar supplied with 1.5 mm H2O2. Fig. S3 Appressorial marker induction and penetration efficiency of the apB73 mutant compared with SG200. (a) Representative photograph showing the analysed filaments on an infected maize leaf. For infection, two strains (SG200AM1‐RFP and SG200ΔapB73‐AM1) at an optical density at 600 nm (OD600) of unity were mixed, infected into maize cv. B73 and stained with calcofluor white at 18–20 h post‐infection. SG200AM1 cells appear red (red fluorescent protein, RFP) and blue (calcofluor white stain). SG200AM1 cells, after induction of the appressorial marker, appear blue/red and green (green fluorescent protein, GFP). SG200ΔapB73‐AM1 strains appear blue or green/blue after induction of the appressorial marker. Bar, 10 µ [file MPP-17-1467-s001.docx]

**Supporting Information**

Article title: Characterization of ApB73, a virulence factor important for colonization of *Zea mays* by the smut *Ustilago maydis*

Authors: Alexandra Stirnberg, Armin Djamei

Article acceptance date:

**The following Supporting Information is available for this article:**

**
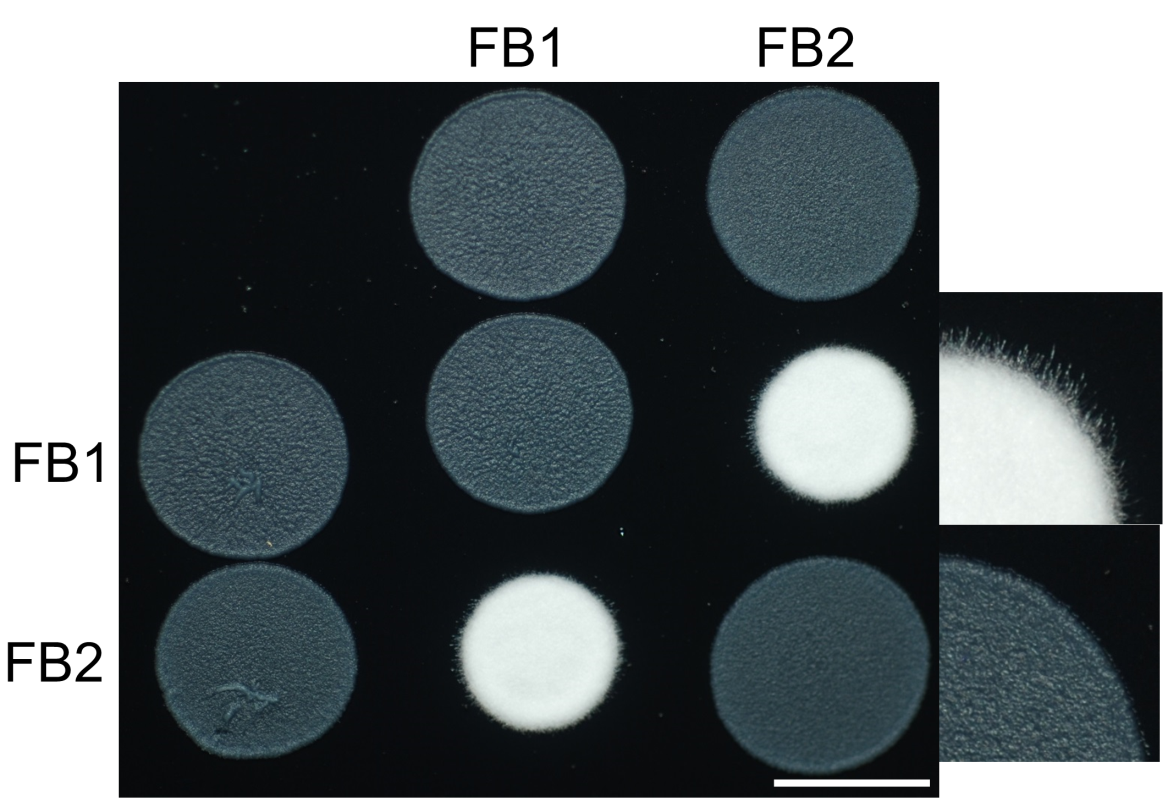
**

**Fig. S1 Filament induction on charcoal plates after mating wildtype strains FB1 and FB2.**

*U. maydis* pre-cultures of the two wildtype strains FB1 and FB2 were grown to an OD_600_ of 1.0 and individually or as a mixture dropped on charcoal plates. The two strains, FB1 and FB2, have compatible mating types that allow them to mate (recognizable by the fuzzy/more white colony morphology), whereas strains with the same mating type cannot mate when mixed together resulting in no filament formation. Picture was taken 24 hours after dropping. Bar = 0.5 cm.


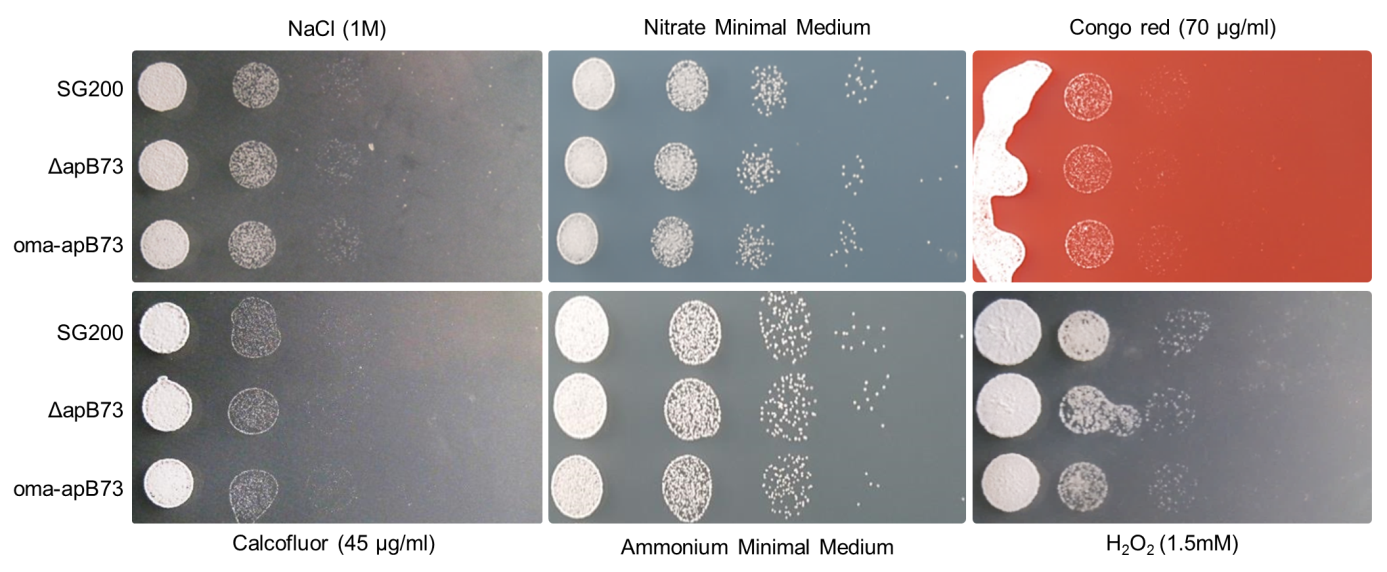
**Fig. S2 Effect of ApB73 on growth under stress conditions.**

Growth of *U. maydis* SG200, SG200ΔapB73 and SG200ΔapB73-P_oma_-apB73 on media providing different stresses. First, *U. maydis* pre-cultures were grown to an OD_600_ of 1.0. Cells were washed in water and 10 fold serial dilutions were prepared. From each solution, 7 µL were dropped on the different media. Pictures were taken 48 hours later. Media used from top left to bottom right: CM agar containing 1M NaCl; Nitrate Minimal Medium; CM agar supplied with Congo red (70 µg/ml); CM agar supplied with Calcofluor (45 µg/ml); Ammonium Minimal Medium; CM agar supplied with 1.5 mM H_2_O_2_.


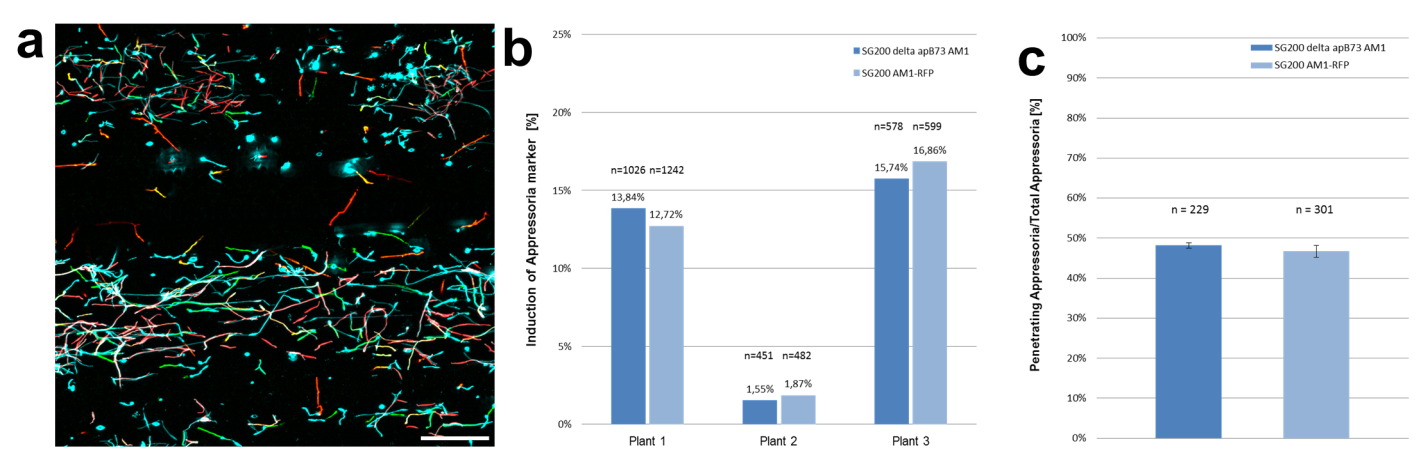


**Fig. S3 Appressoria marker induction and penetration efficiency of the *apB73* mutant compared to SG200.**

**(a)** Representative picture showing the analysed filaments on an infected maize leaf. For the infection two strains (SG200AM1-RFP and SG200ΔapB73-AM1) of OD_600_ = 1 were mixed, infected into maize cv. B73 and stained with Calcofluor white 18-20 hours post infection. SG200AM1 cells appear red (RFP) and blue (Calcofluor white stain), SG200AM1 cells with induced appressoria marker will appear blue/red and green (GFP), SG200ΔapB73-AM1 strains look blue or green/blue after induction of the appressoria marker. Bar = 10µm. **(b)** Comparison of the induced appressoria marker in SG200AM1-RFP or SG200ΔapB73-AM1. Three independent plants were analysed, as efficiency greatly varies depending on the analysed area and plant. Experiment was repeated two times with similar results. **(c)** Penetration efficiency of the formed appressoria in SG200AM1-RFP or SG200ΔapB73-AM1. Penetrated hyphae expressing cytoplasmic GFP cannot be stained by Calcofluor white allowing the quantification of penetration efficiency (ratio of green vs. blue/green hyphae).


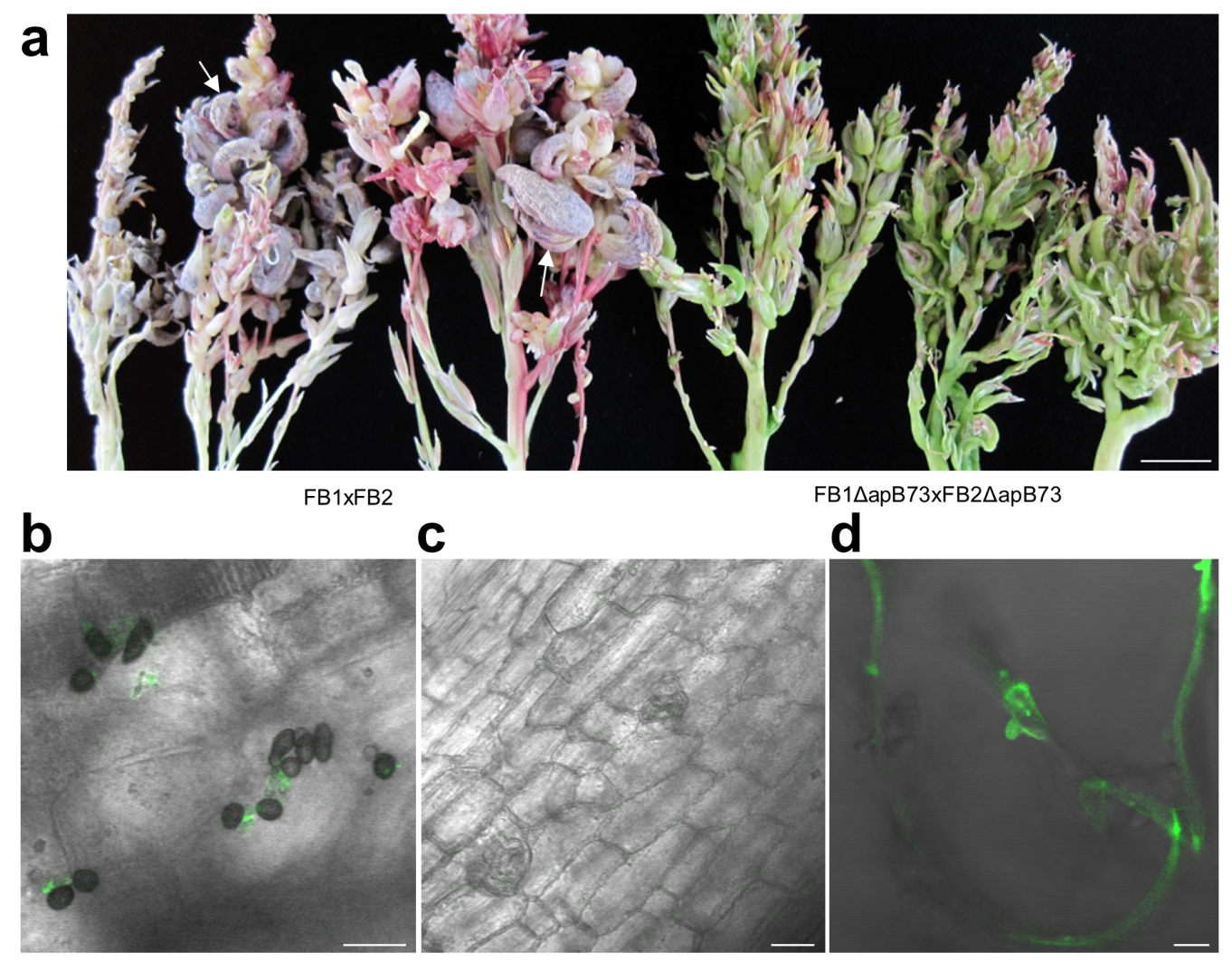


**Fig. S4 Spore formation is impaired after infection with FB1ΔapB73xFB2ΔapB73 on maize cv. Gaspe Flint. (a)** Tassel phenotype of maize plants cv. Gaspe Flint infected with either FB1xFB2 or FB1ΔapB73xFB2ΔapB73. Representative tassels were chosen to illustrate spore development. Picture was taken 18 dpi. White arrows indicate black teliospores. Bar = 1 cm. **(b-d)** Microscopic analysis of infected tassels 18 days post infection labelled with WGA AlexaFluor 488. Bar = 20µm. **(b)** Germinating spores observed in tassel infected with FB1xFB2. **(c)** Tumorous tissue without detectable *U. maydis* cells observed on tassels of plants infected with FB1ΔapB73xFB2ΔapB73. **(d)** Fungal hyphae of *U. maydis* on tassels infected with FB1ΔapB73xFB2ΔapB73.


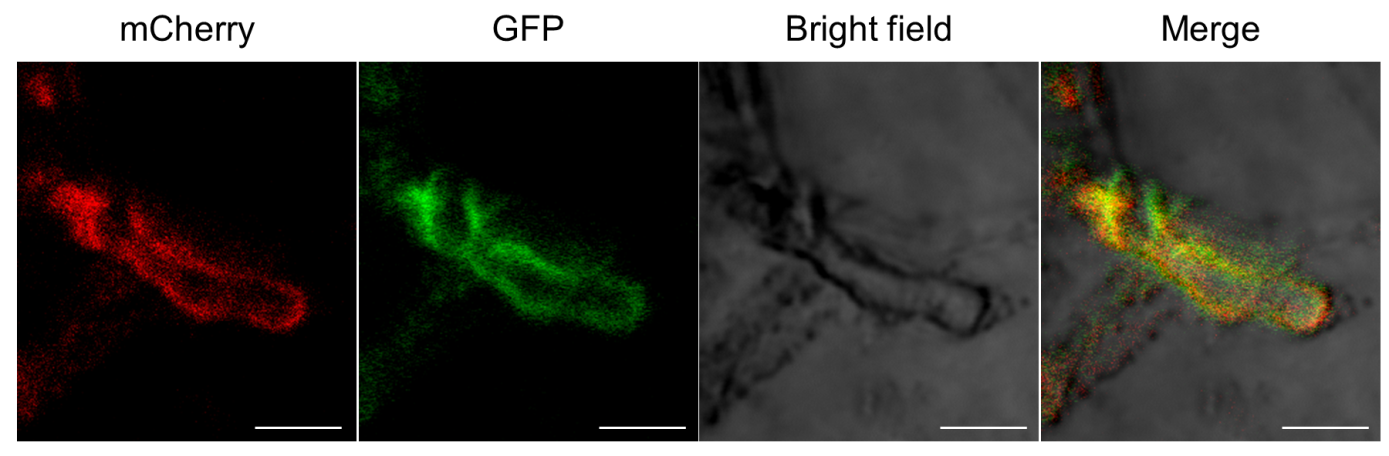


**Fig. S5 Localization of Pit1-GFP and ApB73-mCherry-HA in infected maize leaves.**

Seven day old maize seedlings of cv. B73 were infected with a ΔapB73 strain co-expressing ApB73-mCherry-HA and Pit1-GFP under the native ApB73 promoter. Fluorescence was observed by confocal microscopy three days post infection. Bar = 5 μm.

**
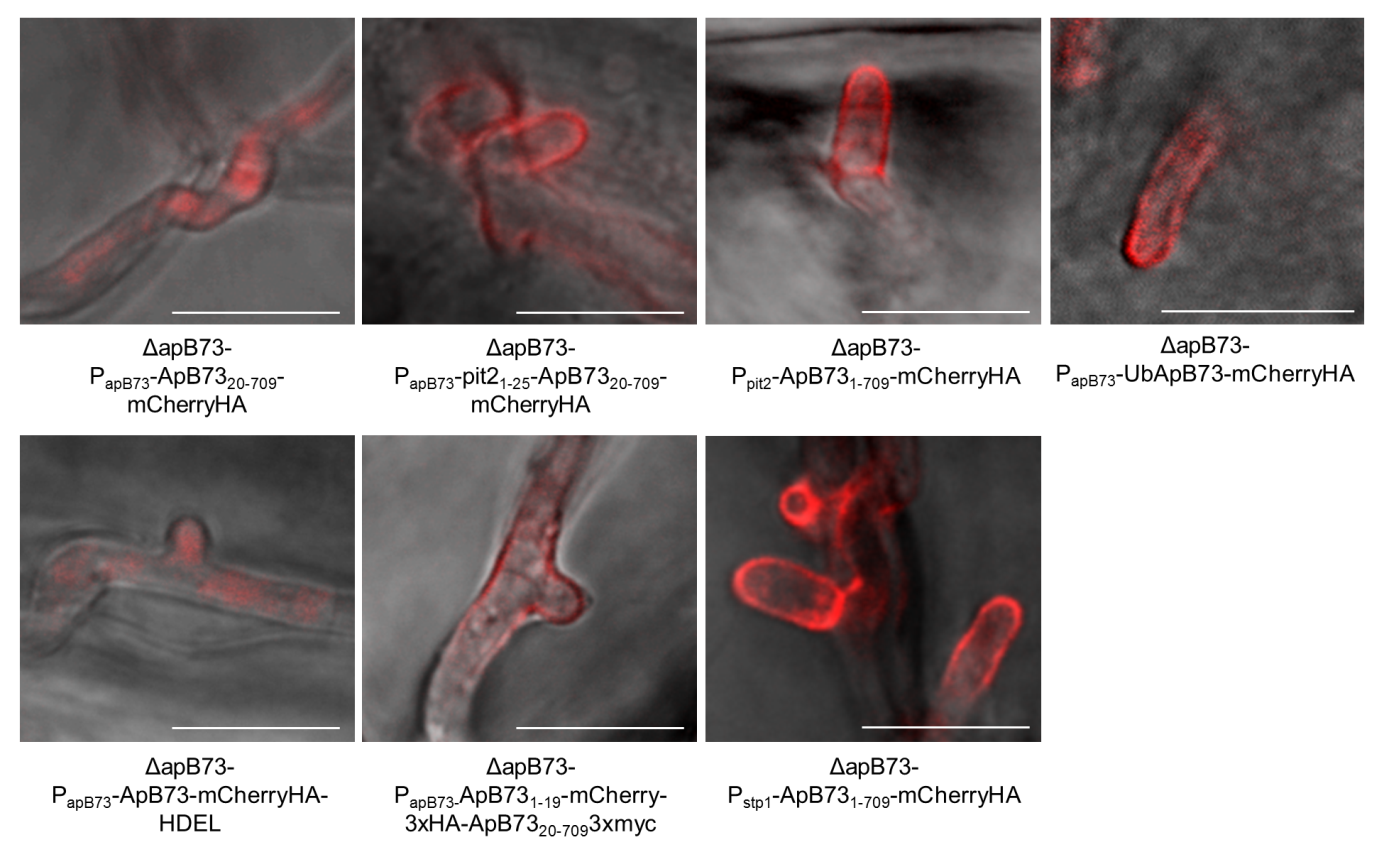
**

**Fig. S6 Microscopy of infected maize leaves to show expression and localization of used fusion proteins.**

Seven day old maize seedlings of cv. B73 were infected with ΔapB73 strains expressing either ApB73_20-709_-mCherryHA, Pit2_1-25_-ApB73_20-709_-mCherryHA, ApB73-mCherry-HDEL, UbApB73-mCherryHA or ApB73_1-19_-mCherry-3xHA-ApB73_20-709_-3xmyc under the native ApB73 promoter. Additionally, it shows maize tissue infected with ΔapB73 strains expressing ApB73-mCherry-HA under either the *pit2* or *stp1* promoter. Fluorescence was observed three to five days post infection. Bar = 10 μm.

**
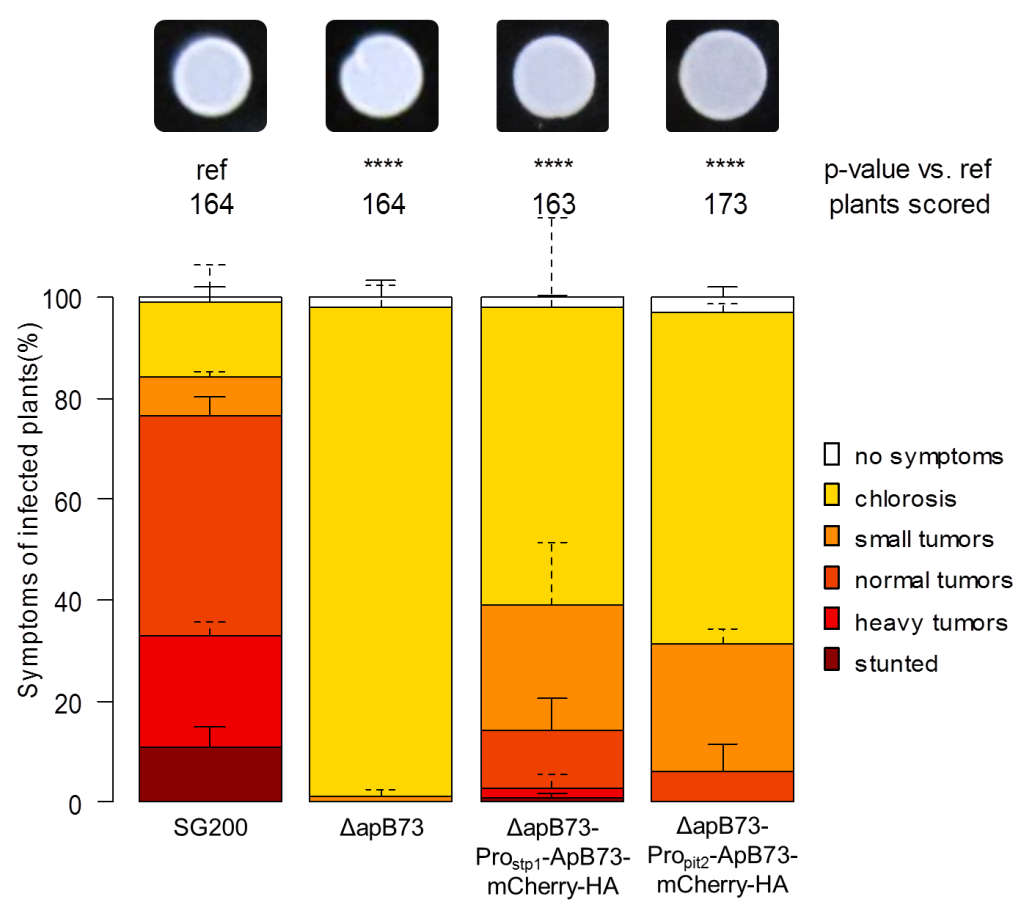

Fig. S7 Virulence assay using promoters strongly upregulated during biotrophy.**

Disease rating of ApB73 expressed under different promoters in the ΔapB73 mutant. The mutant was transformed with a construct expressing ApB73-mCherry-HA under two biotrophy specific promoters. The used promoters express the effector genes *stp1* and *pit2* (Doehlemann *et al.*, 2009; Schipper, 2009). Seedlings of maize cv. B73 were infected seven days after germination and scored for symptoms 12 dpi. In the top row, pictures of the respective strain are shown after growing on filamentation inducing charcoal plates for 24h. Disease scores are shown on the right. Mean values of three independent infections are depicted and the total number of infected plants is indicated above the respective columns. Mean and standard deviation of relative counts from replicates are displayed. For clarity only positive error bars are shown. P-values were calculated by Fisher’s exact test. Multiple testing correction was done using Benjamini Hochberg procedure. ****, P<0.0001.

**Doehlemann G, van der Linde K, Aßmann D, Schwammbach D, Hof A, Mohanty A, Jackson D, Kahmann R. 2009.** Pep1, a Secreted Effector Protein of *Ustilago maydis*, Is Required for Successful Invasion of Plant Cells. *PLOS Pathogens* **5**(2): e1000290.

**Schipper K. 2009.** *Charakterisierung eines Ustilago maydis Genclusters, das für drei neuartige sekretierte Effektoren kodiert.* Philipps-Universität Marburg.

**
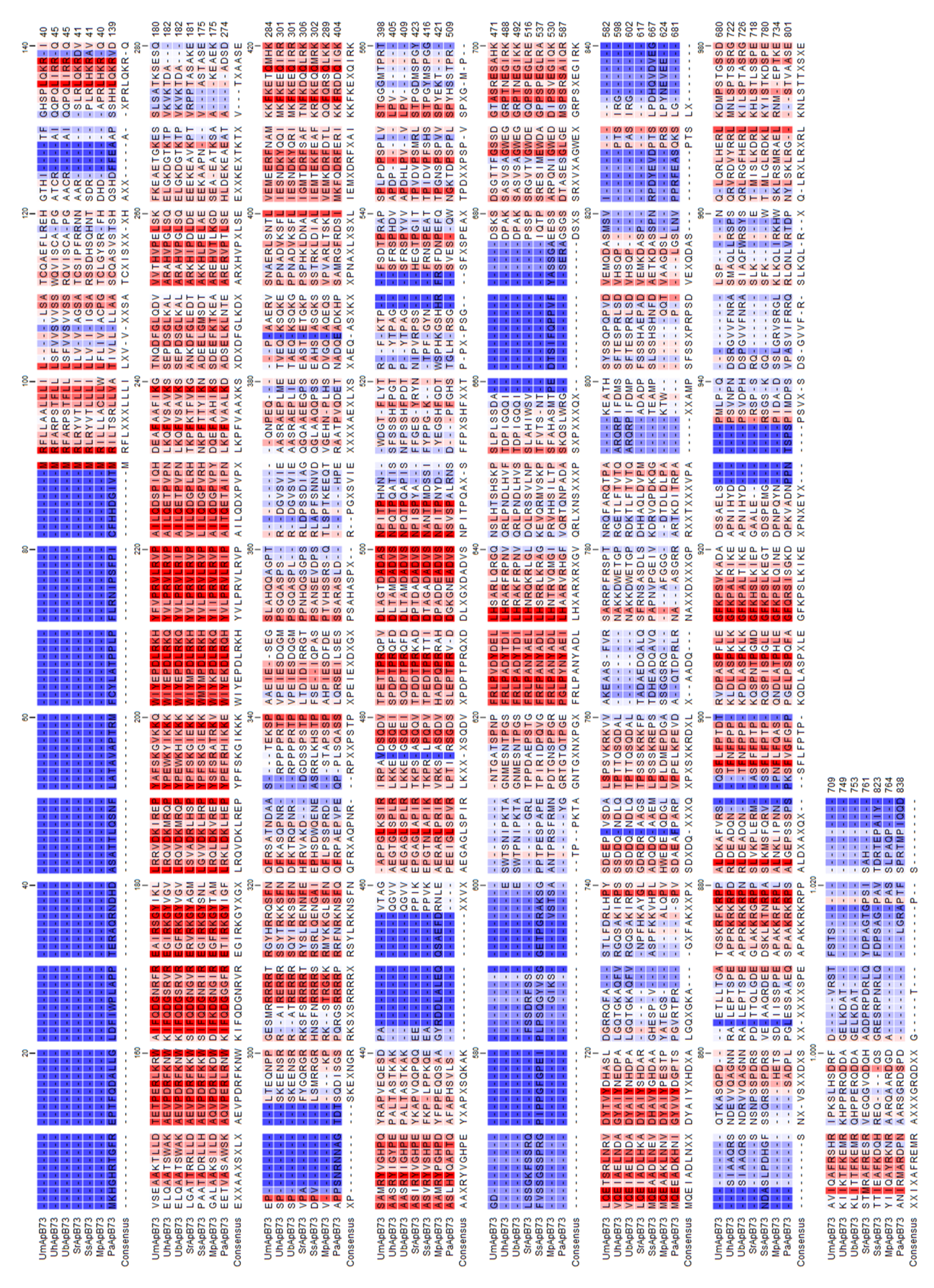
Fig. S8 Amino acid sequence alignment of ApB73 orthologs from different smuts.**

Alignment sequences of the full length proteins from *U. maydis* (UmApB73), *U. bromivora* (UbApB73), *U. hordei* (UhApB73), *S. reilianum* (SrApB73), *S. scitamineum* (ScApB73), *M. pennsylvanicum* (MpApB73) and *P. aphidis* (PaGApB73) were obtained from public databases. Sequence alignment was generated by the multiple sequence alignment program CLUSTAL O in CLC main bench using default parameters. Colour scheme shows consensus strength with red representing the highest and blue the lowest values.
